# Resolving noise–control conflict by gene duplication

**DOI:** 10.1371/journal.pbio.3000289

**Published:** 2019-11-22

**Authors:** Michal Chapal, Sefi Mintzer, Sagie Brodsky, Miri Carmi, Naama Barkai

**Affiliations:** Department of Molecular Genetics, Weizmann Institute of Science, Rehovot, Israel; University of Bath, UNITED KINGDOM

## Abstract

Gene duplication promotes adaptive evolution in two main ways: allowing one duplicate to evolve a new function and splitting ancestral functions between the duplicates. The second scenario may resolve adaptive conflicts that can rise when one gene performs different functions. In an apparent departure from both scenarios, low-expressing transcription factor (TF) duplicates commonly bind to the same DNA motifs and act in overlapping conditions. To examine for possible benefits of this apparent redundancy, we examined the Msn2 and Msn4 duplicates in budding yeast. We show that Msn2,4 function as one unit by inducing the same set of target genes in overlapping conditions. Yet, the two-factor composition allows this unit’s expression to be both environmentally responsive and with low noise, resolving an adaptive conflict that limits expression of single genes. We propose that duplication can provide adaptive benefit through cooperation rather than functional divergence, allowing two-factor dynamics with beneficial properties that cannot be achieved by a single gene.

## Abstract 2

Gene expression tuning is limited by a conflict between expression plasticity and expression noise. This study shows that duplicated genes can resolve this conflict by functioning as one unit with expression control that is both environmentally responsive and low-noise, revealing a new adaptive benefit of duplication.

## Introduction

The number of transcription factors (TFs) expressed in eukaryotes positively correlates with genome size and organism complexity, ranging from approximately 50 in obligate parasites to >1,000 in high eukaryotes [1]. Gene duplication plays a major role in this evolutionary expansion [2,3], as is evident from the fact that the majority of TFs belong to just a few DNA-Binding Domain (DBD) families, and the number of eukaryotic DBD families per species is constant, regardless of the genome size or the number of genes [1]. Understanding the adaptive forces that promote duplication-dependent expansion of TFs is of great interest.

Gene duplication can promote evolution by allowing one of the duplicates to adopt a novel function while the second duplicate maintains the ancestral function. More often, however, the two duplicates do not gain a new function but rather lose complementary subsets of ancestral functions [4,5]. In addition to explaining duplicate maintenance, subfunctionalization can promote adaptive evolution by resolving adaptive conflicts [6,7]. Indeed, optimizing a dual-function protein is often constrained by conflicting requirements imposed by the different functions: a mutation that favors one function can perturb the other function, presenting an adaptive conflict that only upon duplication is resolved to allow further optimization.

In the context of TFs, duplication may allow one factor to acquire a new set of target genes (neofunctionalization). Alternatively, the ancestral targets could split between the duplicates (subfunctionalization). In both scenarios, duplicate divergence would increase and refine the regulatory logic. Previous studies exemplified both scenarios [8–10], but whether they are relevant for the majority of TF duplicates remained unclear.

Budding yeast provide a convenient platform for studying the adaptive roles of TF duplicates. The yeast lineage underwent a Whole Genome Duplication (WGD) event about 100 million years ago [11], which was a result of an interspecies hybridization [12]. While most duplicates generated in this event were lost, about 10% were retained, among which TFs are over-represented. Many of the retained TF duplicates show little signs of divergence in their DBD, bind the same DNA motifs (S1 and S2 Figs), and regulate similar cellular functions, suggesting at least partial redundancy.

Msn2 and Msn4 are a case in point. Previous studies established that the two factors induce an overlapping set of environmental stress response genes [13–15] but also suggested some differences in response kinetics of individual targets. We decided to revisit this analysis using the higher experimental resolution now possible to systematically characterize target divergence under a range of conditions. Our results, however, reinforced the conclusion that the two factors regulate the same set of target genes, translocate to the nucleus with the precise same dynamics, and contribute to stress protection.

Our search for differences between the duplicates pointed us to a different aspect of transcription regulation: the challenge cells face when attempting to minimize noise in gene expression. As a stochastic process, transcription is subject to random variations (noise) [16,17]. Noisy expression is deleterious when affecting genes that require precise tuning [18], such as dosage-sensitive genes [19,20], but can become beneficial when enabling processes not possible by deterministic dynamics [21–23]. Accordingly, noise levels vary greatly between genes [24]. Yet, the ability to tune expression noise through changes in gene promoter is limited. In particular, it is well-documented that genes that are readily regulated by environmental signal also show a high level of expression noise [16,25–27]. This observation is rationalized in two main ways. First, a gene that is regulated by a large number of factors, as required for tuning gene expression with environmental signal, will also show corresponding sensitivity to stochastic variations in its regulators. Second, promoter structures allowing for dynamic response are different from those that encode for constant expression and are therefore associated with increasing noise. For example, flexible promoters bind nucleosomes more loosely and uniformly compared to stable promoters, possibly introducing a nonlinear competition between TFs and nucleosomes. Thus, while coding for low-noise expression is possible, it comes at the cost of lowering the dynamic range over which expression can be changed by regulatory signals.

It was suggested that gene duplication can relieve the coupling between expression noise and plasticity [25]. Our study shows that this is indeed the case for Msn2,4. Following duplication, Msn2 expression became highly stable. It now shows limited responsiveness to environmental conditions and is subject to low expression noise. By contrast, Msn4 expression accentuated the environmental-responsive expression of the unduplicated homolog. This resulted in an overall expression of the Msn2,4 unit that is responsive to the environment yet at the same time maintains low noise expression at the basal, uninduced state. We provide evidence that this expression tuning is phenotypically adaptive and define the genetic changes that correlates with the change in gene responsiveness and noise. Our results suggest that duplicates can promote adaptive evolution not only through functional divergence, as suggested by the neo- or subfunctionalization models, but also through effective cooperation. Through cooperation, the functional unit adopts two-factor dynamics with emergence beneficial properties that cannot be achieved using a single gene.

## Results

### Low-noise Poisson distribution of MSN2 expression in individual cells

**Fig 1:**
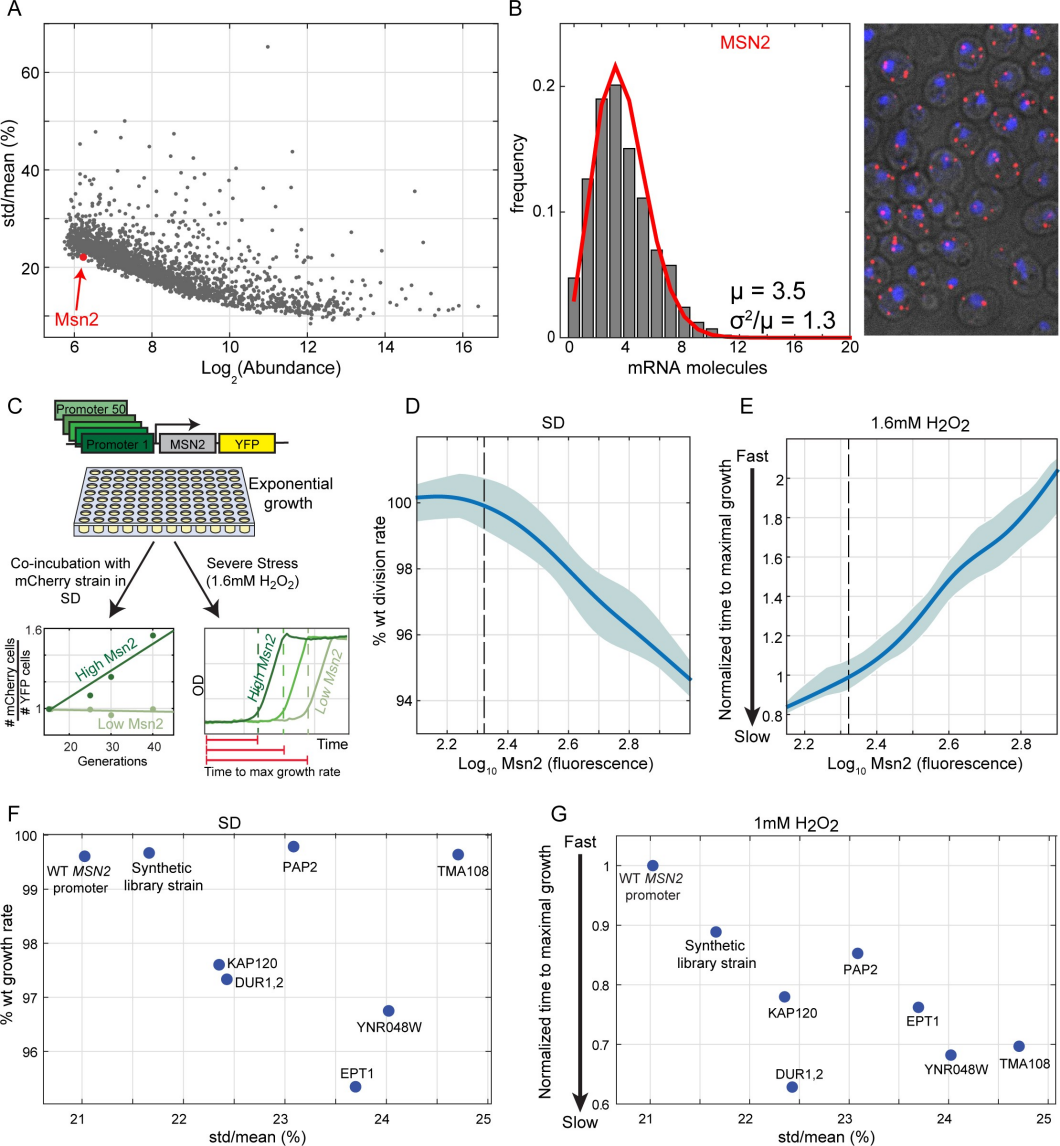
Tuning of Msn2 expression in rapidly growing cells. (A) Cell-to-cell variability of Msn2-GFP is the lowest of all equally abundant proteins: shown are the noise versus abundance data of approximately 2,500 GFP-fused proteins (data from Newman and colleagues [24]). Msn2 is shown as a red dot. Msn4-GFP was not detected. (B) Low-noise (Poisson) distribution of *MSN2* in individual cells: *MSN2* expression levels were measured using smFISH. (Left) *MSN2* mRNA counts distribution, quantified in >650 single cells. Red line represents Poisson fit to the data. (Right) Fixed cells labeled with *MSN2* mRNA in red and DAPI staining in blue, in a maximal *z*-projection image. (C–E) Msn2 expression increases stress protection but slows growth in the absence of stress: we generated a library of 50 strains with *MSN4* deletion and Msn2-YFP expressed under different synthetic promoters (from Keren and colleagues [32]), spanning a range of expression values (C, Materials and Methods). This library was used to measure the effect of Msn2 expression level on growth rate and stress protection. Growth rates were measured using a sensitive competition assay and are shown in (D). Stress protection was measured by subjecting exponentially growing cells to H_2_O_2_ (1.6 mM) and identifying the time at which growth was first detected by continuous OD measurements (E). Shown are the median of all strains and repeats in solid line and 25th–75th percentiles in the shaded areas. Dashed lines indicate WT Msn2 level. (F–G) Noisy Msn2 expression decreases stress protection and growth rate: we generated six strains with Msn2-YFP expressed under different promoters, which control genes with noisier expression than Msn2 but have a similar mean abundance (mean abundance in S5 Fig), and deletion of *MSN4*. These strains, together with an additional strain from the synthetic library (C), were used to measure growth rates (F) and stress protection (G) as described in (D,E) as a function of Msn2 expression noise. Promoter names are indicated in the figure. The raw data for (B) are available in S1 Data, for (D,F) in S2 Data, and for (E,G) in S3 Data. GFP, green fluorescent protein; OD, Optical Density; std, standard deviation; WT, wild type; YFP, yellow fluorescent protein.

Msn2 and Msn4 are TF duplicates that regulate the stress response in budding yeast [13,28]. Stress genes show a noisy expression [29], and we were therefore surprised to observe that Msn2 is expressed at very similar amounts across individual cells. In fact, of the 250 genes with the closest mean abundance to Msn2, only one was less noisy, as quantified in a study surveying >2,500 GFP-fused proteins [24] (Fig 1A). Using single-molecule Fluorescent In Situ Hybridization (smFISH) [30], we found that the number of *MSN2* transcripts in individual cells is well-described by a Poisson distribution, as expected when individual mRNA transcripts are produced and degraded at constant rates [29,31] (Fig 1B and S3 Fig). This distribution represents the lower limit of gene expression noise, obtained in the absence of regulation or other noise-amplifying processes [31].

### Increasing Msn2 expression promotes stress protection but reduces cell growth rate

Low expression noise characterizes genes coding for essential functions or components of large complexes [29,33], for which expression tuning is beneficial [33–35]. By contrast, Msn2 is not essential, does not participate in large complexes, and is mostly inactive in rich media. To examine whether and how Msn2 expression level impacts cell fitness, we engineered a library of strains expressing Msn2 at gradually increasing amounts using synthetic promoters [32]. This library was generated on an *MSN4*-deletion background to eliminate possible compensation effects by Msn4. Measuring growth rates of the library strains using a sensitive competition assay (Fig 1C), we found that decreasing Msn2 expression to below its wild-type levels and down to a complete deletion had no detectable effect on growth rate within the resolution of our assay (0.5%). By contrast, growth rate decreased upon increasing Msn2 abundance (Fig 1D and S4 Fig). Next, we tested the effect of Msn2 levels on the ability to proliferate in harsh stress by incubating the library cells in high H_2_O_2_ concentrations (Fig 1E and S4 Fig). Here, increasing Msn2 levels was beneficial: cells that expressed high levels of Msn2 resumed growth faster than low-expressing ones. Therefore, increasing Msn2 expression better protects cells against stress but reduces their growth rate. An optimal Msn2 level is therefore desirable to balance the need for rapid growth and stress protection, explaining the requirement for low-noise tuning of its gene expression.

To measure directly the phenotypic effect of noisier Msn2, we selected six promoters that are expressed at similar levels to Msn2 but show a higher expression noise and swapped the endogenous *MSN2* promoter with these selected promoters. We swapped the promoters on a background strain with *MSN4* deletion and Msn2-YFP tag, allowing us to measure the mean and noise expression of Msn2 by flow cytometer. We measured growth rates of these strains using a sensitive competition assay (Fig 1F) and found that four of the noisy promoters decreased the growth rate to below its wild-type levels. Next, we tested the effect of the noise on the ability to proliferate in harsh stress by incubating the cells in high H_2_O_2_ concentrations (Fig 1G). We found that as Msn2 expression noise increases, the cells resumed growth more slowly. We therefore concluded that low-noise tuning in Msn2 expression is adaptive and beneficial.

### *MSN4* expression is environmentally sensitive and high-noise

**Fig 2:**
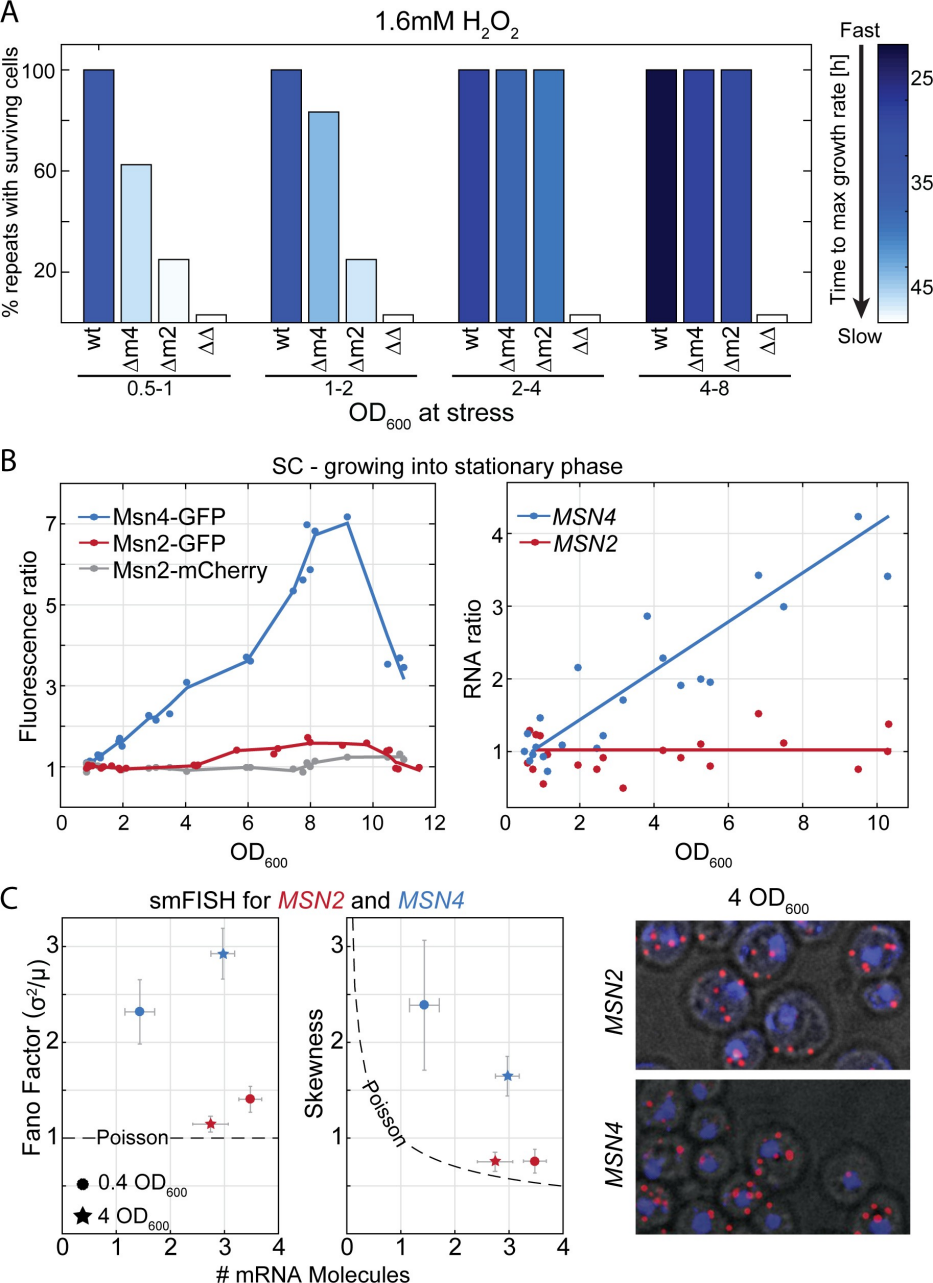
Msn4 expression and its contribution to stress preparation increases as cells exit exponential growth. (A) The contribution of Msn2 and Msn4 to stress preparation changes along the growth curve: cells at different stages along the growth curve (see S6 Fig for growth curve in rich media) were diluted into media containing 1.6 mM H_2_O_2_ and were followed by continuous OD measurements to define the time at which growth was first detected. Shown is the percent of repeats with surviving cells of each strain in different cell densities and the time to resume growth (color-coded). (B) Msn4 expression increases along the growth curve in protein and transcript levels, while Msn2 expression remains stable: samples were taken from cells growing along the growth curve. Expression was measured using fluorescent protein fusion (B, left) and transcription profiles (B, right). Shown is the ratio between each measurement to the low OD measurement. (C) *MSN4* expression is noisy, while *MSN2* expression follows the Poissonian variance: mRNA molecules of *MSN2*,*4* were counted in >4,000 single cells with smFISH in exponentially growing cells (circles) and at OD_600_ = 4 (stars). Shown are the mean number of molecules at the x-axis and the Fano factor (left) and skewness (middle) of the mRNA distribution at the y-axis. Dashed line represents the Poisson distribution parameters. (Right) smFISH imaging examples. The raw data for (A) are available in S3 Data, for (B) in S4 Data, and for (C) in S1 Data. GFP, green fluorescent protein; OD, Optical Density; SC, synthetic complete; smFISH, single-molecule Fluorescent In Situ Hybridization; WT, wild type.

The tradeoff between rapid growth and stress preparation depends on their relative contribution to population fitness, which is a function of growth conditions. For example, when growth conditions are optimal, maximizing division rate dominates, but when nutrients become limiting, protecting against stress becomes increasingly important. Consistent with this, as cells approached stationary phase, they became better protected and resumed growth faster following H_2_O_2_ exposure (Fig 2A).

If Msn2 expression is evolutionarily optimized to account for both rapid growth and stress protection, its expression should be changed in conditions that modify their relative contribution to fitness. We therefore expected Msn2 expression to change, for example, along the growth curve, increasing as cells approach stationary phase. This, however, was not the case. Although Msn2 contributed to stress protection at all densities, its expression remained constant throughout the growth curve (Fig 2B).

Msn4, the Msn2 duplicate, is also a stress genes activator [14,28]. Msn4-GFP was undetectable in reported measurements [36,37], suggesting that its expression level is low during rapid growth. We reasoned that Msn4 expression might increase along the growth curve to account for the changing interplay and promoter stress protection. This was indeed the case: Msn4 expression increased with cell density (Fig 2B). This higher expression was accompanied by increased contribution to stress protection, as was measured by introducing H_2_O_2_ to strains with *MSN2* deletion in different cell densities (Fig 2A). Consistent with the control–noise tradeoff described above, this dynamic regulation of *MSN4* was accompanied by high expression noise, which significantly exceeded the Poissonian variance (Fig 2C).

### Msn2 and Msn4 colocalize to the nucleus with the same dynamics in individual cells

**Fig 3:**
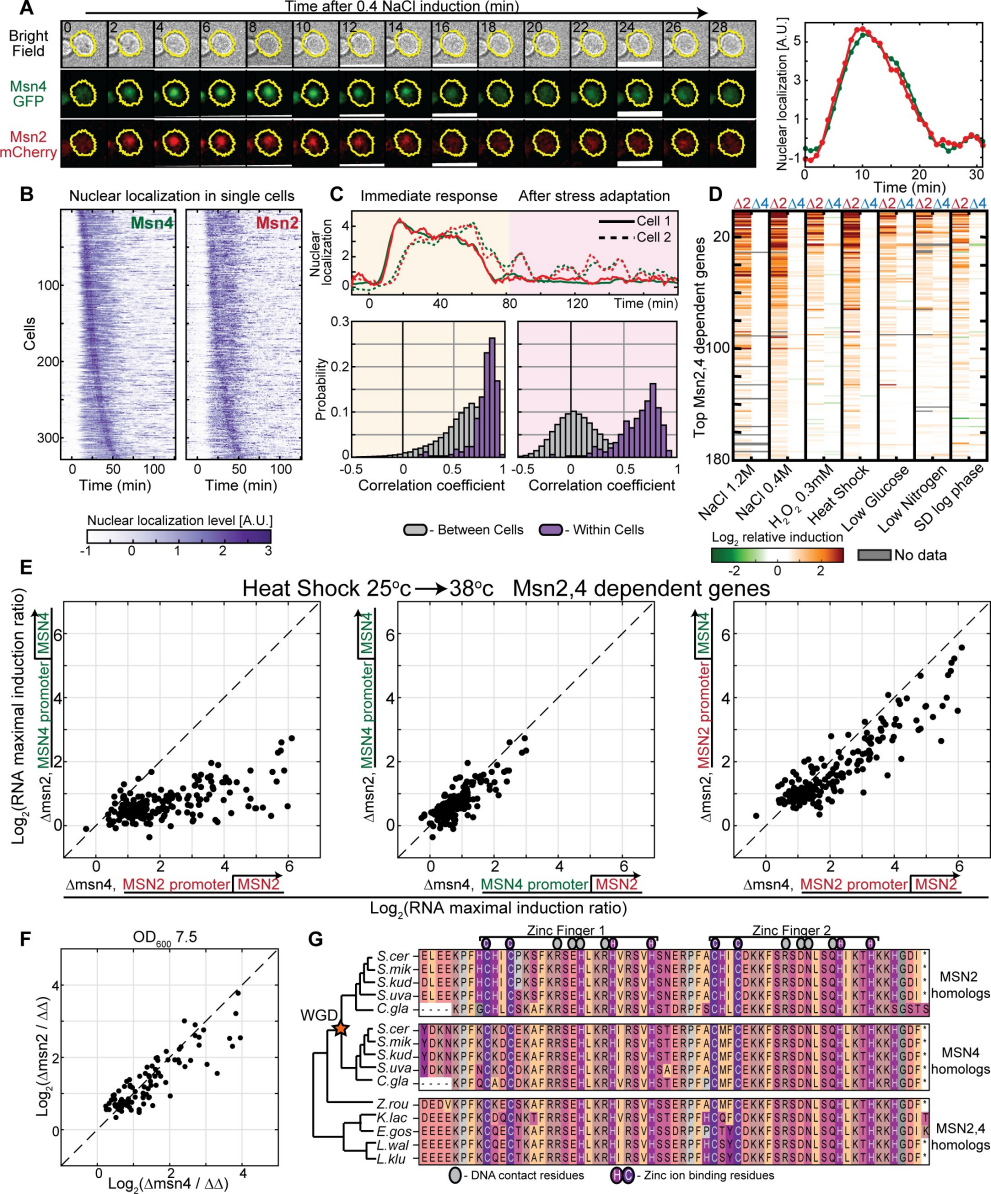
Redundancy in Msn2 and Msn4 activity. (A–C) Single cells expressing Msn4-GFP and Msn2-mCherry were visualized using microfluidics-coupled live microscopy. Both proteins were readily visualized when cells were first cultured at intermediate or high OD (because Msn4 is undetectable in low ODs when cells grow exponentially). Cells were tracked as they were exposed to 0.4 M, 1.2 M, or 1.4 M NaCl. Cells were segmented, and the nuclear localization of both proteins was quantified. A representative cell in time in three channels and a quantification of nuclear localization levels of Msn2 (red) and Msn4 (green) are shown in A. Temporal traces of 328 single cells in 1.2 M NaCl, ordered in both columns by the time of Msn4-GFP nuclear localization, are shown in (B) (0.4 M and 1.4 M NaCl in S8 Fig). Correlations between the individual traces of Msn2 versus Msn4 nuclear localization levels in single cells were calculated. Distributions of the correlation coefficients within the same (purple) or in different (gray) cells are shown in C, separately comparing the immediate response (left) and the longer-time dynamics (right). (D) Stress response in rapidly growing cells depends on Msn2 but not Msn4: exponentially growing cells were exposed to the indicated stresses. Genome-wide transcription profiles were measured at 3-minute time resolution following stress induction for the first 60 minutes and 10-minute for the next 30 minutes. The stress response of each gene was summarized by its integrated (log2) change over the time course. The experiment was repeated in wild-type cells, single-deleted cells (Δmsn2, Δmsn4), and double-deleted cells (Δmsn2Δmsn4). Shown are the differences between gene induction of the wild-type versus the single-deletion strains (Δmsn2 or Δmsn4 at the left/right column, respectively). 180 genes are shown, selected and ordered by the average ratio (over all conditions) between wild-type induction and the double *MSN2* and *MSN4* deletion strain induction. These genes contain stress-induced modules defined by other studies (S10 Fig). (E) Msn2 and Msn4 induce the same set of target genes: during exponential growth, when Msn2 expression is higher than Msn4, deletion of Msn2 results in a significantly stronger effect on stress gene expression **(**left**)**, but this effect was fully reversed by swapping the Msn2 and Msn4 promoters **(**middle and right**)**. Each dot represents a target gene and its induction ratio between the indicated strain and the double *MSN2* and *MSN4* deletion strain. (F) Msn2 and Msn4 in high OD (7.5): when both factors are expressed, stress genes are induced equally. Each dot is an induced target gene. (G) Msn2 and Msn4 bind DNA through a highly conserved DBD: Alignment of Msn2 and Msn4 DBDs and their homologs in 10 species of the *Ascomycota* phylum that diverged before or after the WGD event (star). Colors indicate amino acid residue types. The raw data for (B,C) are available in S5 Data and for (D–F) are available at SRA under BioProject PRJNA541833. A.U., arbitrary unit; DBD, DNA-Binding Domain; GFP, green fluorescent protein; OD, Optical Density; SRA, Sequence Read Archive; WGD, Whole Genome Duplication.

Our results above show that Msn2 and Msn4 contribute additively to stress protection, and we further verified this by replacing each of these proteins by its paralog (S7 Fig). This additive contribution could result from regulation of the same set of genes or through induction of a distinct set of targets. Similarly, it could respond to the same or to different sets of post-translational factors. Since activated Msn2,4 translocate to the nucleus [38,39], we defined their activation pattern by following their nuclear translocation dynamics using fluorescent-tagged proteins (Fig 3A). In response to osmotic stress, the two factors translocated to the nucleus within minutes, showing precisely the same kinetics within individual cells (Fig 3B and 3C, yellow shade, and S8 Fig). Similarly, translocation of the two proteins also remained highly synchronized within individual cells during the stochastic pulsing following stress [39,40] (Fig 3C, pink shade). Deletion of *MSN2* did not affect the dynamics of its duplicate Msn4 (S9 Fig).

### Msn2 and Msn4 induce the same set of target genes

Next, we examined for differences in Msn2,4 target genes using time-resolved, genome-wide transcription profiling of cells subject to a variety of stresses. In rapidly growing cells, deletion of *MSN2* strongly reduced stress gene induction, while deletion of *MSN4* had little, if any, effect (Fig 3D and S11 Fig). Swapping the *MSN2*,*4* promoters completely reversed the target induction capacity of these factors (Fig 3E and S12B Fig). The identity of the targets remained the same: Msn4 driven by the *MSN2* promoter induced precisely the same targets normally induced by Msn2. The induction of the targets was almost as high as the induction of Msn2, suggesting that most of this effect is governed by the promoter and some minor effect by the induction capacity of Msn4. When tested in conditions in which Msn4 is highly expressed, the two factors induced the same set of genes (Fig 3F and S12 Fig). Since a previous study [41] that followed stress induction of individual genes using fluorescence reporters indicated some differences in individual targets dependence on Msn2,4, we examined specifically the genes reported to be differently regulated. However, none of these genes showed any difference in their Msn2,4 dependency in any of the six conditions for which we performed tight time-course measurements (S13 Fig). While some of the reported differences may be due to strain or condition differences, we attribute them mostly to differences in the resolution of our measurements (see S1 Note for discussion).

To further corroborate these results, we used Chromatin Endogenous Cleavage sequencing ChEC-seq) [42] to measure the genome-wide binding profiles of Msn2,4. The binding profiles of the two factors were indistinguishable (S14 Fig). This identity of Msn2,4 targets is consistent with the high conservation of their DNA binding domains (Fig 3G), and identity of their in vitro DNA binding preferences[43] (S15 Fig). We conclude that Msn2,4 proteins are co-regulated by the same signals and, at the same kinetics, activate the same set of target genes with the same kinetics, essentially functioning as one TF.

### Differential architecture of the *MSN2*,*4* promoters explains the differences in their expression flexibility and noise

**Fig 4:**
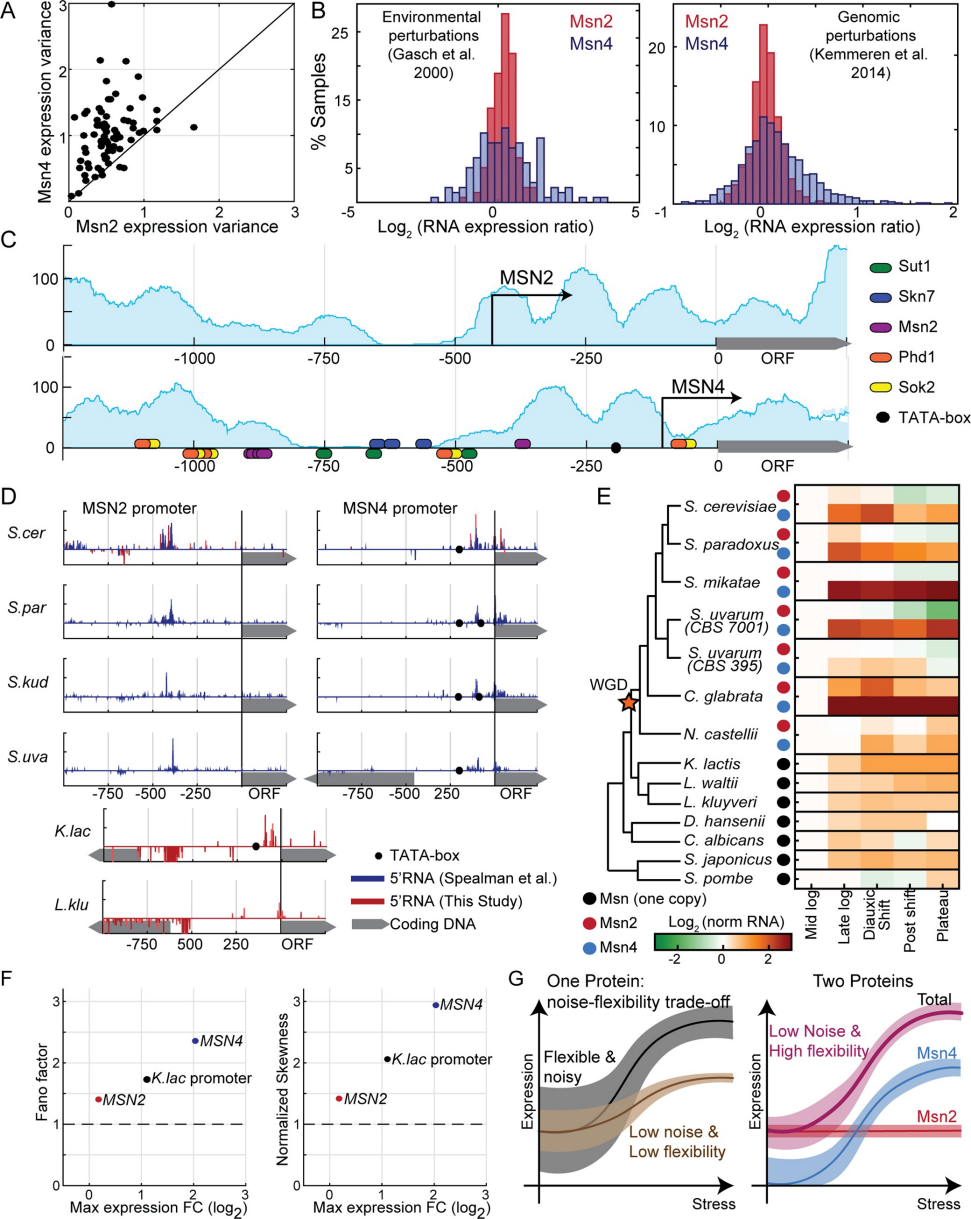
*MSN2* shifted its TSS and gained a stable expression pattern in species that diverged from *Saccharomyces cerevisiae* following WGD. (A–B) Three data types were considered. First, we downloaded >230 mRNA expression data sets available in SPELL [44] and compared the variance of *MSN2* and *MSN4* expression in each data set with more than 20 samples (A, each data set is a dot). Second, we compared the distribution of *MSN2* and *MSN4* expression levels in two large data sets, representing multiple stress conditions [13] (B, left) or gene deletions [45] (B, right). (C) The *MSN2* promoter displays properties of the stable, low-noise type, while *MSN4* promoter conforms to the flexible noisy type: the pattern of nucleosome occupancy along the two promoters as defined by Weiner and colleagues [46] is shown in blue shade. Arrows represent TSS positions, as defined by Park and colleagues [47]. Ellipses denote TF binding sites as defined by MacIsaac and colleagues [48]. TATA box (black circles) is defined as TATA[AT]A[AT]. (D) *MSN2* promoter displays an uncharacteristically long 5′ UTR that is conserved in all species that diverged after the WGD event: shown are the promoter maps of *MSN2*,*4* homologs in the indicated species. mRNA 5′ end mapping data from Spealman and colleagues [49] are shown in blue, mRNA 5′ end from this study in red. TATA box is defined as in C. (E) *MSN2* homologs are stably expressed along the growth curve, while *MSN4* homologs show the flexible expression of the single MSN homologs found in species that diverged from *S*. *cerevisiae* prior to the WGD event: shown are expression levels of the *MSN2*,*4* homologs in all indicated species, in 5 time points along the growth curve. Data from Thompson and colleagues [50]. (F) Expression of the *Kluyveromyces lactis MSN2*,*4* homolog shows intermediate flexibility and noise. On the x-axis, the maximal fold change expression of *MSN2*, *MSN4*, and the *K*. *lactis* homolog (data from Thompson and colleagues [50]) is shown. y-Axes show attributes of the expression distribution measured by smFISH, in *MSN2*, *MSN4*, and *MSN2* in *S*. *cerevisiae* driven by the promoter of the *K*. *lactis* homolog. Shown are the Fano factor (left) and the skewness of the distribution normalized to the skewness of a Poisson distribution with the same mean as the data (right). (G) Model: duplication of Msn2,4 resolved conflict between environmental responsiveness and noise: single genes whose expression is sensitive to environmental conditions but will suffer from high noise in nonstressed conditions, limiting the ability to precisely tune intermediate expression levels while maintaining environmental-responsive expression. Gene duplication can resolve this conflict. See text for details. The raw data for (F) are available in S1 Data. smFISH, single-molecule Fluorescent In Situ Hybridization; SPELL, Serial Pattern of Expression Levels Locator; TF, transcription factor; TSS, Transcription Start Site; WGD, Whole Genome Duplication.

Msn2 expression is stable along the growth curve, while Msn4 is strongly induced. To examine whether this differential dynamics is specific to these conditions or is a more general property of the two genes, we surveyed a data set composed of thousands of transcription profiles [13,44,45]. Expression of *MSN2* showed little variability in all reported conditions, while *MSN4* was variable (Fig 4A and 4B). Expression of *MSN2* and *MSN4* therefore conforms to the general tradeoff between expression noise and regulatory control: Msn2 is stable across conditions and shows low cell-to-cell variability (noise), while Msn4 expression readily responds to environmental signals and is noisy.

Previous studies defined promoter types that encode for flexible and noisy or stable and low-noise expression [51–53]. Flexible promoters tend to contain a TATA box and bind nucleosomes immediately upstream to their Transcription Start Site (TSS), while stable promoters lack a TATA box and display a Nucleosome-Free Region (NFR) upstream of their TSS. Consistent with their differential flexibility, we find that the *MSN4* promoter contains a TATA box, binds nucleosomes around its TSS, and includes a large number of TF binding sites. By contrast, the *MSN2* promoter does not contain a TATA box, displays an NFR immediately upstream of the TSS, and is largely devoid of TF binding sites (Fig 4C; data from [46–48]).

When aligned by their coding frames, the nucleosome patterns along the upstream regions of *MSN2* and *MSN4* promoters are highly similar. However, the location of the TSS is different: in *MSN4*, the TSS is positioned approximately 105 bp away in a region that is nucleosome occupied, while in *MSN2*, the TSS is significantly further upstream and located on the border of an NFR. The resulting 5′ UTR of *MSN2* is exceptionally long (approximately 430 bp in length; longer 5′ UTRs are found in only 2% of *S*. *cerevisiae* genes; data from [47]).

To examine whether the differential architectures of the *MSN2*,*4* promoters are indeed responsible for their differential expression flexibilities, we first checked whether the region regulating *MSN4* expression is the NFR region, predicted to bind multiple TFs. This was indeed the case because deleting this region practically abolished Msn4 induction along the growth curve (S16 Fig). Furthermore, replacing this region in the *MSN4* promoter with the corresponding region from *MSN2* promoter, which includes an NFR and the *MSN2* TSS, increased *MSN4* expression and reduced its noise (S17 Fig). Therefore, as predicted, this promoter region accounts for the differential expression characteristics of *MSN2* and *MSN4*.

### *MSN2* TSS was shifted following the WGD event

Msn2,4 were generated in the WGD event approximately 100 million years ago [11], which was a result of an interspecies hybridization [12], and were retained in all WGD species tracing to this event. To examine whether the differential promoter structure of *MSN2*,*4* is conserved in other WGD species, we used available 5′ RNA data [49] and further profiled TSS positioning in these species. The TSS positions of the *MSN2* and *MSN4* homologs were conserved in all post-WGD species (Fig 4D). Sequence analysis indicated that also the TATA box was conserved in all *MSN4* homologs but absent from all *MSN2* homologs (Fig 4D). We next profiled 5′ RNA in two non-WGD species. The transcript of the single *MSN* homolog has a short 5′ UTR, similar to that of *MSN4*. This pattern of conservation is consistent with a scenario in which the stable *MSN2* promoter evolved from an ancestral flexible promoter through a shift in the TSS to a distant, TATA-lacking position at the boundary of a nearby NFR.

### *MSN4* accentuated the environmentally responsive but noisy expression of the non-WGD homolog while *MSN2* gained a stable, low-noise expression

To examine whether the differential expression flexibility of *MSN2*,*4* is also conserved in the other post-WGD species, we used available expression data [50] of 13 yeast species along their growth curves. In all post-WGD species, *MSN4* expression increased along the growth curve while *MSN2* expression remained stable (Fig 4E). The single *MSN* homolog in the non-WGD species showed a moderate induction along the growth curve, with a dynamic range that was larger than that of *MSN2* but lower than that of *MSN4* (Fig 4E).

To examine whether this intermediate regulation is also reflected in the expression noise of these proteins, we introduced the *MSN* promoter from *K*. *lactis*, a non-WGD species, into *S*. *cerevisiae* upstream of the *MSN2* ORF and measured expression noise using smFISH. As predicted, this promoter showed an intermediate noise level that was higher than *MSN4* but lower than *MSN2* (Fig 4F). In fact, when plotted on the noise–control curve, the three promoters all fell on the same line, consistent with same-proportion change in noise and dynamic range of regulated expression. Therefore, our analysis suggests that *MSN2* gained its stable, low-noise expression following the duplication event, likely by shifting its TSS, while *MSN4* accentuated the regulated expression of the ancestral factor, likely through the acquisition of new binding sites for TFs, increasing its dynamic range and expression noise.

## Discussion

Taken together, we find that Msn2,4 function as one unit to regulate stress response genes. The two paralogs are translocated to the nucleus with the same kinetics, bind and regulate the same set of target genes, and contribute additively to stress protection. Consistent with previous studies [15,54], we also observe a more severe phenotype of Msn2 deletion when stressing rapidly growing cells, but we now show that this results from the low expression of Msn4 under these conditions and not from a differential function.

What limits replacement of Msn2,4, in at least some species, by a single factor of a more refined transcriptional control? Our data show that Msn2,4 function as one unit whose expression is both environmentally responsive and low-noise (Fig 4G), thereby resolving an inherent conflict that limits the tuning of individual gene expression. Msn2 provides the low-noise basal expression, whereas Msn4 is induced when additional amounts are needed. It is difficult to predict the evolutionary forces that promoted the evolution of Msn2,4 expression features, but since the Msn duplication traces to the WGD event, it is tempting to propose that its new expression characteristics were driven by the shift in metabolism: rapidly growing non-WGD species respire, while WGD species ferment. Following this metabolic change, genes needed in respiring cells may shift from being constitutively expressed to being Msn-dependent, as was indeed reported [55]. We propose that changes in the identity of Msn2,4-dependent genes accentuated its phenotypic effects on growth and drove selection for increased precision of Msn2 expression.

Gene duplication is a major source of evolutionary innovation [4,5] that greatly contributes to the expansion of transcription networks [2,3]. A surprisingly large fraction of TF duplicates, however, retained a conserved DBD and bind to the same DNA motif (S1 and S2 Figs). Whether these duplicates bind and regulate the same set of targets is not known, but the case of Msn2,4 suggests that at least a fraction of them do. Such an apparent redundancy does not comply with the accepted models of neo- or subfunctionalization explaining duplicate advantage. Our study supports a third model whereby duplicates with redundant biochemical properties realize dynamic properties that are not possible or are difficult to achieve using a single factor. In the case of Msn2,4, duplication resolved a conflict between regulatory control and noise. In fact, duplicated genes were reported previously to show higher regulatory plasticity and to gain more TATA boxes since the WGD event as compared to singletons[25]. This suggests an additional case of a relief of the noise–control conflict in other duplicates. In other cases, interactions between the factors may define a circuit with dynamic properties not implementable by a single gene [56–58]. Further studies will define the relative contribution of such circuit-forming mechanisms in explaining the retention of TFs or other duplicates.

## Materials and methods

### Strains

All strains used in this study and their genotypes are listed in S1 Table. All the strains were constructed by standard genetic methods and were validated by PCR and/or sequencing of the relevant DNA. The strains with the duplication of Msn2 and Msn4 (S1 Table, 31–32) were generated by PCR duplication procedure, described by Huber and colleagues [59].

### smFISH

For each gene (*MSN2* and *MSN4*), a set of 48 probes was generated as described in Raj and colleagues [30]. The probes were designed by the online program Stellaris Probe Designer from Biosearch Technologies (Novato, CA, USA) and were ordered with a fluorescent dye CAL Fluor Red 590 (Biosearch Technologies). Probe sequences are listed in S2 and S3 Tables.

Cells were grown overnight in synthetic complete (SC) medium at 30°C and constant shaking. Then diluted to reach the wanted cell densities after approximately 12 hours. Cells were fixated, prepared, and hybridized as described in Rahman and colleagues [60].

Images were acquired with a 100× 1.4 oil UPLSAPO objective, using an Olympus IX83 based Live-Imaging system equipped with CSU-W1 spinning disc (sCMOS digital Scientific Grade Camera 4.2 MPixel, Oxford Instruments, Abingdon, UK). For each sample, 4–6 different positions were chosen. In each position, three-channel *Z*-stacks images were taken with a step size of 200 nm for a total of >6 μm: bright-field image, 488 nm laser with 100 mW; DAPI image, 405 nm laser with 120 mW and exposure time of 250 ms; mRNA image, 561 nm laser with 100 mW and exposure time of 1,000 ms. Each z-plane image was of size 2,048 × 2,048 pixels.

*Single-molecule quantification*. Cells were segmented using a modification of a custom MATLAB (The MathWorks, Natick, MA, USA) software [61]. In this modification, cell centers were defined manually using the bright-field images, and cell borders were found automatically. mRNA counts were then performed for each cell based on the custom-made MATLAB software from Raj and colleagues [30].

### Stress experiments for RNA-seq levels

In these experiments, we used the WT strains, the single msn2 or msn4 deletion strains, and the double msn2 and msn4 deletion strain. Some of the experiments were also done with the strains with swapped promoters: *MSN2* ORF under *MSN4* promoter with a deletion of *MSN4* and the opposite, *MSN4* ORF under *MSN2* promoter with an *MSN2* deletion.

*Growth conditions*. Cells were grown overnight in rich medium—YPD or SC medium at 30°C (unless otherwise noted)—and constant shaking, then cells were diluted and exponentially grew for 6–8 hours before introducing the stress:

*Oxidative stress*. Cells were grown continuously in 30°C. H_2_O_2_ was added to a final concentration of 0.3 mM.

*Heat shock*. Cells were grown continuously in 25°C, then cell culture was moved to a new flask located inside a bath orbital shaker (Cat. WBT-450; MRC, London, UK) preheated to 37°C. It took less than 90 s for the culture to reach 37°C.

*Glucose limitation*. Cells were grown in SC medium with 2% glucose (Sigma-Aldrich, St. Louis, MO, USA). Then, cells were washed twice and resuspended in SC with 0.1% glucose. Samples were taken before the washes (2% glucose), after every wash, and for the next 100 min.

*Osmotic shock*. Cells were grown continuously in 30°C. 4 M NaCl solution was added to the culture to a final concentration of 0.4 M or 1.2 M.

*Low nitrogen*. Cells were grown in SC medium with 2% glucose (Sigma-Aldrich). Then cells were washed twice and resuspended in nitrogen-depleted medium (0.67% Yeast Nitrogen Base without amino acids and ammonium sulfate [Bacto-YNB], 2% glucose, 0.05 mM ammonium sulfate, 20 mg/l uracil, 20 mg/l histidine, 100 mg/l leucin, 20 mg/l methionine).

*Growth into stationary phase*. Cells were grown in SC in 30°C without changing the media.

### RNA sample collection, extraction, and sequencing

Cells were grown overnight to stationary phase and then diluted in 100 ml to reach OD_600_ of 0.2–0.4 after 6–8 hours in constant shaking. A sample for time-point zero reference was taken, and then we introduced a stress perturbation as described above. For the growing into stationary phase experiment, a sample of 1 ml was taken every 20 or 30 minutes. For all other conditions, a sample of 1.5 ml was collected every 3 minutes for the first hour and every 10 minutes for an additional half/one hour. Samples were immediately centrifuged for 40 s in 13,000 rpm. The supernatant was removed, pellets were frozen in liquid nitrogen and stored at −80°C until RNA preparation.

RNA was extracted using a modified protocol of the nucleospin 96 RNA kit (Macherey-Nagel, Duren, Germany). Specifically, cell lysis was done in a 96 deep-well plate by adding 450 μl of lysis buffer containing 1 M sorbitol (Sigma-Aldrich), 100 mM EDTA, and 0.45 μl lyticase (10 IU/μl). The plate was incubated at 30°C for 30 minutes in order to break the cell wall and then centrifuged for 10 minutes at 2,500 rpm, and supernatant was removed. From this stage, extraction proceeded as in the protocol of nucleospin 96 RNA kit, only substituting β-mercaptoethanol with DTT.

For all samples sequenced by the Illumina HiSeq 2500 (Illumina, San Diego, CA, USA), RNA libraries were created as follows: fragmented, poly(A)-selected RNA extracts of approximately 200 bp size were reverse-transcribed to cDNA using barcoded poly(T) primers. cDNA was amplified and sequenced with an Illumina HiSeq 2500 using a primer complementary to the opposite adaptor to the poly(A).

For all samples sequenced by the Illumina NextSeq 500, RNA libraries were created as follows: poly(A) RNA was selected by reverse transcription with a barcoded poly(T) primer. The barcoded DNA–RNA hybrids were pooled and fragmented by a hyperactive variant of the Tn5 transposase. Tn5 was stripped off the DNA by treatment with SDS 0.2%, followed by SPRI beads cleanup, and the cDNA was amplified and sequenced with the Illumina NextSeq 500.

### Processing and analysis of RNA-seq data

We mapped 50-bp reads of the RNA-seq of every sample to the *S*. *cerevisiae* genome (R64 in SGD) using bowtie (parameters:–best -a -m 2 -strata -5 10). After alignment to the genome, samples that had less than 150,000 reads were discarded from the analysis in order to prevent an artificial enrichment for highly expressed genes. The expression at those time points was calculated as the mean between the two closest time points in the time course. For every sequence, we normalized for PCR bias using the unique molecular identifier (UMI), scoring each position on the genome by the unique number of UMIs it had out of all possible UMIs. For each gene, we summed all the reads aligned to 400 bp upstream its 3′ end to 200 bp downstream in order to get the total expression of that gene. Reads that were aligned nonuniquely were split between the aligned loci according to the ratio of all other uniquely mapped reads in these regions. The number of reads for each sample was normalized to 10^6^.

### Msn2-Promoter library preparation

We used 140 synthetic promoters from Keren and colleagues [32] pooled together and transformed them to replace the native *MSN2* promoter in a strain with Msn2 tagged with YFP and deleted of msn4. We collected approximately 200 colonies after the transformation and measured YFP fluorescence with a flow cytometer (BD LSRII system from BD Biosciences, San Jose, CA, USA). We picked 50 strains that spanned the expression range and were highly similar between the different repeats.

### Growth experiment in harsh stress

*MSN2 promoter library strains*. We grew the cells to stationary phase in SC media in a 96-well plate under constant shaking and 30°C. Next, we diluted the cells with fresh SC media in deep-well plates with one glass bead in each well to generate proper shaking so they would reach the wanted OD in the next morning. Then, right before stressing the cells, we took 150 μl to measure ODs (using infinite200 reader; Tecan Inc., Männedorf, Switzerland) and 100 μl to a flow cytometer to measure Msn2-YFP fluorescence.

*Other strains*. We grew cells overnight to stationary phase in SC media and constant shaking at 30°C. Next, we serially diluted the cells with fresh SC media in a 96-well plate to reach sequential different ODs in the next morning. Then, right before stressing the cells, we took 150 μl to measure ODs (using infinite200 reader, Tecan Inc.) and diluted the cells to the same density.

*Stress and growth measurements*. We added 30 μl of growing cells to plates with 120 μl of H_2_O_2_. We inserted the plates into an automated handling robot (EVOware, Tecan Inc.) in which cells were grown in an incubator under constant shaking and 30°C. The robot was programed to take the plates out of the incubator every 30 or 45 minutes, vortex the plates, and measure the OD (using infinite200 reader, Tecan Inc.). Experiments lasted for approximately 70 hours. (An EVOware script for this experiment can be provided upon request.)

*Growth analysis*. Data from the growth measurements were parsed and processed. Time to exponential growth was calculated as the time of the maximal slope of the OD measurements (versus time). We calculated the median time and the standard deviation of the repeats.

### Competition experiment in SC media

Cells were grown ON to stationary phase in SC, then diluted and grown for approximately 8 hours in exponential growth. Each strain was then coincubated with WT-mCherry strain at 30°C. WT initial frequency was approximately 50%. Approximately every 8 hours, cells were diluted with fresh SC media so they would grow exponentially at all times. In addition, a sample was taken to measure OD and to a flow cytometer to measure frequencies of each population. Flow cytometry measurements and analysis were done using the BD LSRII system (BD Biosciences). Flow cytometry was conducted with excitation at 488 nm and emission at 525 ± 25 nm for GFP samples. For mCherry markers, excitation was conducted at 594 nm and emission at 610 ± 10 nm. The number of generations was calculated from the dilution factor. Percent of WT division rate was calculated as previously described in Kafri and colleagues [62].

### Msn2,4 protein expression by flow cytometry

For this experiment, we used a strain with Msn2 tagged with GFP and a strain with Msn4 tagged with GFP and Msn2 tagged with mCherry. We grew the cells overnight in 5 mL SC media at 30°C and constant shaking to reach the stationary phase, then we diluted the cells to reach OD_600_ approximately 0.4 after approximately 8 hours. Next, we serially diluted the cells in a 96-well plate by diluting each column to the next one in a 1:1 ratio with SC media, ending up with 120 μl in each well, and a 1:2 ratio of cells in adjacent columns. After overnight incubation in 30°C and constant shaking, we measured the fluorescence using flow cytometer.

Flow cytometry measurements and analysis were done using the BD LSRII system (BD Biosciences). Flow cytometry was conducted with excitation at 488 nm and emission at 525 ± 25 nm for GFP samples. For mCherry markers, excitation was conducted at 594 nm and emission at 610 ± 10 nm. The average number of cells analyzed was 50,000. For the samples with high OD, 100 μl of DDW was added to the sample before reading it in the FACS.

We calculated cell density using the flow cytometer parameters and output. This measure was calculated as following: $\frac{N}{V}C$ where R = flow rate (μl /s), T = total flow time (s), V = R*T = total volume read (μl), N = number of cells read (cells), C = dilution fix constant (values are either 1 for no dilution with DDW or 1.8333 for samples that were diluted with 100 μl DDW).

We filtered G1 cells similarly to how it was described in Hornung and colleagues [26]. Specifically, we filtered by the width size measure FSC-W, which has a bimodal distribution that corresponds to cells in G1 (smaller) and cells in G2/M (bigger). Next, we filtered outliers by two area measures, FSC-A and SSC-A. We used linear regression to describe FSC-A with SSC-A and removed cells that were far from the regression line. We then applied linear regression to describe SSC-A with FSC-A and removed outliers in a similar manner.

In order to eliminate the background fluorescence, we used a linear regression model that predicts background fluorescence. The independent variables were size parameters (FSC-W and SSC-W) and the cell density of the population. The dependent variable was the background fluorescence (GFP/mCherry). The model was trained on BY4741 cells with no fluorescent markers, then used to predict background in the other strains. Predicted background was subtracted from observed fluorescence for each cell.

### Msn2 protein expression noise by flow cytometry

To measure the noise in the strains expressing Msn2-YFP, we grew the cells overnight in 5 mL SC media at 30°C and constant shaking to reach the stationary phase. Then, we diluted the cells to reach OD_600_ approximately 0.4 after approximately 8 hours. We measured fluorescence using a flow cytometer as described in the previous part. We then filtered the cells and calculated the noise of the population as described in Hornung and colleagues [26].

### Time-lapse microscopy experiment

We used a strain with both Msn2-mCherry and GFP-Msn4 and a strain with GFP-Msn4 and a deletion of Msn2. We grew the cells overnight in SC media to reach stationary phase, then diluted them to reach the desired OD_600_ (approximately 7) after approximately 8 hours. When reaching the desired OD, cells were transferred to a microfluidics plate (catalog number: Y04C-02-5PK; MilliporeSigma, Burlington, MA, USA) for haploid yeast cells. We used an ONIX CellAsic microfluidics system, which allows changing the cells’ media at a fast rate in a predefined set time while not interfering with the imaging process. During imaging, after approximately 15 minutes of flowing the original media of the cells, medium with NaCl was added to the cells (0.4/1.2/1.4 M). Two positions were taken for each strain.

*Imaging*. We used a Zeiss AxioObserverZ1 inverted microscope (Carl Zeiss, Oberkochen, Germany) equipped with Hamamatsu Flash4 sCMOS cameras (Hamamatsu, Hamamatsu City, Japan). In every imaging instance (every 1 minute for 4–8 hours), three images were taken for each position: bright-field image, GFP image using GFP filter with 20% intensity of HSP120 V lamp and with exposure time of 200 ms, and mCherry image using mPlum filter with same intensity and exposure as GFP. We used 2 × 2 binning, resulting in 1,024 × 1,024 pixels of image size.

### Processing microscopy images and estimate nuclear localization levels

*Tracking and segmentation*. All images were subsequently analyzed using custom MATLAB software that segments and tracks individual cells along the movie in each bright-field image frame, as previously described [61]. Briefly, cell borders were detected automatically in the last frame. Then, the program goes back to the beginning of the experiment frame by frame and, for each cell in the image, uses the centroid coordinates of the cells from the previous frame. Each centroid is expended until the borders of the cell in the current frame is found. The program also outputs a score for the segmentation that was used to filter out cells with low quality segmentation.

*Image processing*. Median filter: we ran a 3 × 3 median filter on all GFP and mCherry images. Background removal was done by running a mean filter of 50 × 50 on each image, then subtracting the filtered image from the original one. Rare events of missing frames (mainly due to focus issues) were interpolated to be the mean of the two adjacent frames.

*Calculating nuclear localization measure*. Our method uses image filtering with a filter shaped like a nucleus with a radius of 3 pixels. We run the filter on each cell GFP/mCherry track and then find the maximal coordinate of the filtered cell image, defining it as the center of the hypothetical nucleus. Our measure is the average over the pixels in the hypothetical nucleus divided by the pixel average in the hypothetical cytoplasm. For normalization, we divide each cell’s Msn2/4 nuclear localization in time by the minimal value for this cell. As a result, this method, in fact, gives signal-to-noise ratio. In order to align the GFP and the mCherry tracks together, we used *z*-score normalization (subtracted the mean and divided by standard deviation for each cell).

*Filtering bad cells*. We filtered out “bad” cells in two rounds, once after running segmentation and once after calculating the dynamical attributes. In the first round, we filtered cells that answered one or more of the following conditions: (1) cells with area outside the range defined as *median* ± 3 × mean absolute deviation (MAD) over all cells at least 10% of the time, (2) cells with segmentation score <8 at least 10% of the time, and (3) cells that did not appear from the beginning of the experiment. In the second round, we removed cells with response amplitude below 1.1 or above 6.

### MSN2,4 homolog expression in growth

Data were taken from Thompson and colleagues [50]. For each yeast species in this experiment, 5 time points were measured as a ratio to mid-log sample: lag, late log, diauxic shift, postdiauxic shift, plateau. For each time point, 3 repeats were made. We show the average of the repeats. We exclude the first time point (lag) from the figure because of a large amount of missing data and repeats.

### 5′ mRNA sequencing

Cells were grown overnight to stationary phase in SC media in 30°C and then diluted and exponentially grown for approximately 12 hours in constant shaking. Samples were fixed by mixing them with cold (−80°C) methanol. RNA was poly(A)-selected, reverse-transcribed to cDNA, and barcoded at the 5′ end using Dynabeads Oligo(dT)_25_ magnetic beads (Thermo Fisher Scientific, Waltham, MA, USA). cDNA was pooled and fragmented by a hyperactive variant of the Tn5 transposase. Tn5 was stripped off the DNA by SDS 0.2% treatment, followed by SPRI beads cleanup, and the cDNA was amplified and sequenced with an Illumina NextSeq 500. The number of reads for each sample was normalized, and genomic tracks were created from the sequenced reads, representing the enrichment on each position of the genome.

### ChEC-seq strains

We fused Msn2 or Msn4 to MNase (Amino Acids 83–231) using pGZ108 (pFA6a-3FLAG-MNase-kanMX6). This plasmid was a gift from Steven Henikoff (Addgene plasmid #70231; Watertown, MA, USA).

### ChEC-seq experiment

Cells were grown overnight to stationary phase in SC media in 30°C and then diluted and grown for approximately 15 hours in 30°C and constant shaking until they reached OD_600_ of approximately 4. Then, ChEC-seq was performed as described in Zentner and colleagues [42] with 30 s of activated Mnase, and changes in the ethanolic precipitation (1 hour in −80°C), and SPRI beads size selection (0.8×). Library preparation was performed as describe in Orsi and colleagues [63], with converting the S-300 column cleanup following the phenol-chloroform step to ethanolic precipitation. Libraries were sequenced with an Illumina NextSeq 500.

### ChEC-seq analysis

Reads were mapped to the *S*. *cerevisiae* genome (R64 in SGD) using bowtie2. The first nucleotide of every read was counted as a binding signal. All samples had >10^6^ reads. Each sample was normalized to 10^7^ reads. Promoter length was defined as 700 bp upstream to the TSS or the distance to the upstream transcript (the shorter between these two). Transcription start and end sites were taken from Pelechano and colleagues [64]. For the motif analysis, the average of the sum of signal of each 7-mer appearance (±10 bp) in all of the promoter regions was calculated.

## Supporting information

S1 Fig DNA-binding motifs of duplicated TFs are highly similar. We used available position frequency matrices of all available DNA-binding motifs in YeTFaSCo [65] (“expert collection”) and measured similarity using Tomtom [66]. Here, we show the CDFs of the Q-values similarities between motifs of duplicated TFs (red) and random TFs (blue). CDF, cumulative distribution function; TF, transcription factor; YeTFaSCo, Yeast Transcription Factor Specificity Compendium.
(TIF)

S2 Fig All *S*. *cerevisiae* zinc finger TF duplicates from the WGD event. (Left) Alignment of binding domains of all duplicated pairs. (Right) DNA-binding motifs of the pairs from YeTFaSCo [65]. TF, transcription factor; WGD, Whole Genome Duplication; YeTFaSCo, Yeast Transcription Factor Specificity Compendium.
(TIF)

S3 Fig *MSN2* expression distribution fits a Poisson distribution, while *MSN4* expression is noisier. *MSN2* (left) and *MSN4* (right) expression levels were measured by smFISH at OD_600_ 4, where both TFs showed similar mean expression. Shown are mRNA molecule count distributions. Red lines represent the best Poisson fit to the data. Raw data are available in S1 Data. OD, Optical Density; smFISH, single-molecule Fluorescent In Situ Hybridization; TF, transcription factor.
(TIF)

S4 Fig Growth curves in SC or H_2_O_2_ in Msn2 overexpression or deletion strains. Cells were grown in the indicated condition under constant shaking and 30°C in 96-well plates in an automated handling robot (EVOware, Tecan Inc.). OD was measured automatically approximately every 30 minutes for 65 hours using Infinite200 reader. Raw data are available in S3 Data. OD, Optical Density; SC, synthetic complete.
(TIF)

S5 Fig Mean abundance versus growth rates of strains expressing noisy Msn2-GFP. Noisy Msn2 strains were generated by swapping the endogenous *MSN2* promoter (“source strain”; dark red), with other, noisier gene promoters. Shown are these strains and one strain from the synthetic library strain, as indicated in the legend. Gray shade indicates the synthetic library strains phenotype for a reference (see Fig 1D for details). Raw data are available in S2 Data. GFP, green fluorescent protein.
(TIF)

S6 Fig Growth curve of the WT strains BY4741. Shown are OD measurements on the y-axis (logarithmic scale) as a function of time. Error bars represent standard deviation of 16 repeats. OD, Optical Density.
(TIF)

S7 Fig Msn2 and Msn4 contribution to stress protection is additive. We generated a strain with *MSN2* duplication and msn4 deletion and a strain with *MSN4* duplication and *MSN2* deletion. We measured stress protection by diluting cells at different stages along the growth curve into media containing 1.6 mM H_2_O_2_ and measuring OD continuously to define the time at which maximal growth was first detected. Shown is the time to resume maximal growth, normalized to the time it took the WT strain to resume maximal growth. Red line represents the WT strain. Raw data are available in S3 Data. OD, Optical Density; WT, wild type.
(TIF)

S8 Fig Nuclear translocation of Msn2,4. Single cells expressing Msn4-GFP and Msn2-mCherry fusion proteins were tracked using microfluidics-coupled live microscopy in 0.4/1.2/1.4 M NaCl. (Left) Localization dynamics following exposure to stress is shown as the medians, and the single cell traces are shown as shaded lines. (Right) Individual nuclear localization traces of both Msn2 and Msn4 are shown, with cells in both columns presented in the same order. Raw data are available in S5 Data. GFP, green fluorescent protein; Msn, XXX.
(TIF)

S9 Fig Nuclear translocation of Msn4 in WT cells and cells in which *MSN2* is deleted. (A,B) Localization dynamics following exposure to 0.4/1.2M NaCl is shown as the median. (C,D) Individual traces of Msn4 in WT cells of cells deleted of msn2. Raw data are available in S5 Data. WT, wild type.
(TIF)

S10 Fig Msn2,4-dependent genes. We calculated Msn2,4 dependency score for each gene as the average over all conditions, of ratio between WT induction and the double msn2, msn4 deletion strain induction. The 500 top Msn2,4-dependent genes are ordered by this score. Shown are the scores and an indication if the genes are part of the written published data sets [13,67] (black: gene is part of the group, white: gene is not part of the group). Raw data are available at SRA under BioProject PRJNA541833. SRA, Sequence Read Archive; WT, wild type.
(TIF)

S11 Fig RNA expression in all conditions. Cells were grown to exponential phase; then, at OD_600_ 0.2–0.4, they were exposed to stress. Samples for mRNA measurements were taken every 3 minutes for the first hour after stress induction and every 10 minutes for the next one/half an hour. In addition, we took samples along the growth curve every 20–30 minutes (SC). Raw data are available at SRA under BioProject PRJNA541833. OD, Optical Density; SC, synthetic complete; SRA, Sequence Read Archive.
(TIF)

S12 Fig Msn2 and Msn4 induce the same target genes. (A) Clustering of all genes in all the conditions and repeats that we checked. For each experiment of the stress perturbations, we calculated for each strain the AUC, and for cells growing into the stationary phase, we used expression in different ODs. We then calculated the fold change of WT or single deletions to the double-deletion strain and used these values to cluster genes. (B) Swapping Msn2,4 promoter. Shown is the fold change of gene induction in response to H_2_O_2_ in the indicated strains relative to the double-deletion strain. Each dot represents a gene that was >2-fold higher in the WT then the double deletion. (C) Shown is the fold change of gene-induction different stress conditions in the single-deletion strains relative to the double-deletion strain. Each dot represents a gene that was >2-fold higher in the WT then the double-deletion strain. Raw data are available at SRA under BioProject PRJNA541833. AUC, area under the curve; OD, Optical Density; SRA, Sequence Read Archive; WT, wild type.
(TIF)

S13 Fig Response to stress of reported genes from AkhavanAghdam and colleagues [41]. Plotted are mRNA measurements (from our study) of the response of the four genes reported in AkhavanAghdam and colleagues [41]. Shown are mRNA measurements for the WT, single-, and double-deletion msn2,4 strains in response to various stress conditions. All stresses were introduced to cells growing exponentially (0.2–0.4 OD_600_). In addition, we measured mRNA expression along the growth curve (SD). Dots represent the data measurements, and lines are the smoothed signal. In our high-temporal–resolution data, there is no fundamental difference in Msn2,4 contribution to the response between the first two genes (DSC2, DDR2) and last two genes (SIP18, TKL2) as was reported. In all of these genes, *MSN4*-deleted strains show similar expression and dynamics to the WT strain, but *MSN2*-deleted strains reduce the induction significantly. Raw data are available at SRA under BioProject PRJNA541833. OD, Optical Density; SRA, Sequence Read Archive; WT, wild type.
(TIF)

S14 Fig Msn2,4 prefer the same DNA-binding sequence and the same promotors in vivo. (A) Msn2 and Msn4 binding to all the promotors. Sum of the normalized ChEC-seq signal of each factor measured in cells at OD approximately 4 was calculated for all the promotes in >4 repeats. Shown is the *z*-score of the median of all repeats. Color represents the correlation of Msn2 and Msn4 binding signal on the promoters. (B) Density plot comparing Msn2 and Msn4 in vitro binding to all possible (8,192) 7-DNA base pair sequences. For each 7-mer, the mean signal of all its appearances in all promoters was calculated for Msn2 and Msn4. Shown is the density plot of the *z*-scores of all possible 7-mers. (C) DNA motifs found in our data for Msn2 and MSN4. Raw data are available at SRA under BioProject PRJNA573518. ChEC-seq, Chromatin Endogenous Cleavage sequencing; OD, Optical Density; SRA, Sequence Read Archive.
(TIF)

S15 Fig Msn2,4 prefer the same DNA-binding sequence site in vitro. Density plot comparing Msn2,4 in vitro binding to all possible (32,896) 8-DNA base pair sequences. Data from Siggers and colleagues [43].
(TIF)

S16 Fig *MSN4* promoter regions. We generated five strains with partial *MSN4* promoter by cutting the upstream part of the promoter in the indicated places in the scheme. Shown are median expression levels of Msn4-GFP along the growth curve in the strains with full and partial *MSN4* promoter. The highlighted areas in the scheme show the promoter regions that induce Msn4 at high ODs. Raw data are available in S4 Data. GFP, green fluorescent protein; OD, Optical Density.
(TIF)

S17 Fig *MSN2* NFR and TSS promoter region determines the expression level and noise. (A) A scheme of the strain we used—*MSN4* promoter with a swap with *MSN2 NFR*+*TSS* in the same position. (B) smFISH results of the swapped strain and the WT *MSN2*,*4* in the indicated ODs. Raw data are available in S1 Data. NFR, Nucleosome-Free Region; OD, Optical Density; smFISH, single-molecule Fluorescent In Situ Hybridization; TSS, Transcription Start Site; WT, wild type.
(TIF)

S1 Data smFish experiments data. smFISH, single-molecule Fluorescent In Situ Hybridization.
(XLSX)

S2 Data Competition assays data. (XLSX)

S3 Data Growth in H_2_O_2_. (XLSX)

S4 Data Msn2,4-tagged protein measurements by flow cytometer. (XLSX)

S5 Data Nuclear localization data. (XLSX)

S1 Table Yeast strains used in this study. (DOCX)

S2 Table MSN2 smFISH probes, CAL Fluor Red 590. smFISH, single-molecule Fluorescent In Situ Hybridization.
(DOCX)

S3 Table MSN4 smFISH probes, CAL Fluor Red 590. smFISH, single-molecule Fluorescent In Situ Hybridization.
(DOCX)

S1 Note Discussion about differences between the results of this study and AkhavanAghdam and colleagues [41]. (DOCX)

## Abbreviations

Bacto-YNB: Yeast Nitrogen Base without amino acids and ammonium sulfate
ChEC-seq: Chromatin Endogenous Cleavage sequencing
DBD: DNA-Binding Domain
GFP: green fluorescent protein
MAD: mean absolute deviation
NFR: Nucleosome-Free Region
OD: Optical Density
SC: synthetic complete
smFISH: single-molecule Fluorescent In Situ Hybridization
SPELL: Serial Pattern of Expression Levels Locator
SRA: Sequence Read Archive
TF: transcription factor
TSS: Transcription Start Site
UMI: unique molecular identifier
WGD: Whole Genome Duplication
YFP: yellow fluorescent protein

## References

[1] Charoensawan V, Wilson D, Teichmann SA. Genomic repertoires of DNA-binding transcription factors across the tree of life. Nucleic Acids Research. 2010;38: 7364–7377. 10.1093/nar/gkq617 20675356 PMC2995046

[2] Weirauch MT, Hughes TR. A Catalogue of Eukaryotic Transcription Factor Types, Their Evolutionary Origin, and Species Distribution. Subcel Biochem. 2011;52: 25–73. 10.1007/978-90-481-9069-0_3 21557078

[3] Lambert SA, Jolma A, Campitelli LF, Das PK, Yin Y, Albu M, et al The Human Transcription Factors. Cell. 2018;172: 650–665. 10.1016/j.cell.2018.01.029 29425488 PMC12908702

[4] Conant GC, Wolfe KH. Turning a hobby into a job: How duplicated genes find new functions. Nature Reviews Genetics. 2008;9: 938–950. 10.1038/nrg2482 19015656

[5] Soskine M, Tawfik DS. Mutational effects and the evolution of new protein functions. Nature Reviews Genetics. 2010;11: 572–582. 10.1038/nrg2808 20634811

[6] Hittinger CT, Carroll SB. Gene duplication and the adaptive evolution of a classic genetic switch. Nature. 2007;449: 677–681. 10.1038/nature06151 17928853

[7] Des Marais DL, Rausher MD. Escape from adaptive conflict after duplication in an anthocyanin pathway gene. Nature. 2008;454: 762–765. 10.1038/nature07092 18594508

[8] Voordeckers K, Pougach K, Verstrepen KJ. How do regulatory networks evolve and expand throughout evolution? Current Opinion in Biotechnology. 2015;34: 180–188. 10.1016/j.copbio.2015.02.001 25723843

[9] Pérez JC, Fordyce PM, Lohse MB, Hanson-Smith V, DeRisi JL, Johnson AD. How duplicated transcription regulators can diversify to govern the expression of nonoverlapping sets of genes. Genes & development. 2014;28: 1272–7. 10.1101/gad.242271.114 24874988 PMC4066398

[10] Baker CR, Hanson-Smith V, Johnson AD. Following gene duplication, paralog interference constrains transcriptional circuit evolution. Science (New York, NY). 2013;342: 104–8. 10.1126/science.1240810 24092741 PMC3911913

[11] Wolfe KH, Shields DC. Molecular evidence for an ancient duplication of the entire yeast genome. Nature. 1997;387: 708–713. 10.1038/42711 9192896

[12] Marcet-Houben M, Gabaldón T. Beyond the Whole-Genome Duplication: Phylogenetic Evidence for an Ancient Interspecies Hybridization in the Baker’s Yeast Lineage. Hurst LD, editor. PLoS Biol. 2015;13: e1002220 10.1371/journal.pbio.1002220 26252497 PMC4529251

[13] Gasch AP, Spellman PT, Kao CM, Carmel-Harel O, Eisen MB, Storz G, et al Genomic Expression Programs in the Response of Yeast Cells to Environmental Changes. Silver PA, editor. Molecular Biology of the Cell. 2000;11: 4241–4257. 10.1091/mbc.11.12.4241 11102521 PMC15070

[14] Schmitt AP, McEntee K. Msn2p, a zinc finger DNA-binding protein, is the transcriptional activator of the multistress response in Saccharomyces cerevisiae. Proceedings of the National Academy of Sciences of the United States of America. 1996;93: 5777–82. 10.1073/pnas.93.12.5777 8650168 PMC39137

[15] Estruch F. Stress-controlled transcription factors, stress-induced genes and stress tolerance in budding yeast. FEMS Microbiology Reviews. 2000;24: 469–486. 10.1111/j.1574-6976.2000.tb00551.x 10978547

[16] Elowitz MB, Levine AJ, Siggia ED, Swain PS. Stochastic gene expression in a single cell. Science (New York, NY). 2002;297: 1183–6. 10.1126/science.1070919 12183631

[17] Raser JM, O’Shea EK. Noise in gene expression: origins, consequences, and control. Science (New York, NY). 2005;309: 2010–3. 10.1126/science.1105891 16179466 PMC1360161

[18] Schmiedel JM, Carey LB, Lehner B. Empirical mean-noise fitness landscapes reveal the fitness impact of gene expression noise. Nature Communications. 2019;10: 3180 10.1038/s41467-019-11116-w 31320634 PMC6639414

[19] Lehner B. Selection to minimise noise in living systems and its implications for the evolution of gene expression. Molecular Systems Biology. 2008;4: 170 10.1038/msb.2008.11 18319722 PMC2290932

[20] Metzger BPH, Yuan DC, Gruber JD, Duveau F, Wittkopp PJ. Selection on noise constrains variation in a eukaryotic promoter. Nature. 2015;521: 344–347. 10.1038/nature14244 25778704 PMC4455047

[21] Eldar A, Elowitz MB. Functional roles for noise in genetic circuits. Nature. 2010;467: 167–173. 10.1038/nature09326 20829787 PMC4100692

[22] Raj A, van Oudenaarden A. Nature, Nurture, or Chance: Stochastic Gene Expression and Its Consequences. Cell. 2008;135: 216–226. 10.1016/j.cell.2008.09.050 18957198 PMC3118044

[23] Yaakov G, Lerner D, Bentele K, Steinberger J, Barkai N. Coupling phenotypic persistence to DNA damage increases genetic diversity in severe stress. Nature Ecology & Evolution. 2017;1: 0016 10.1038/s41559-016-0016 28812556

[24] Newman JRS, Ghaemmaghami S, Ihmels J, Breslow DK, Noble M, DeRisi JL, et al Single-cell proteomic analysis of S. cerevisiae reveals the architecture of biological noise. Nature. 2006;441: 840–846. 10.1038/nature04785 16699522

[25] Lehner B. Conflict between Noise and Plasticity in Yeast. Akey JM, editor. PLoS Genetics. 2010;6: e1001185 10.1371/journal.pgen.1001185 21079670 PMC2973811

[26] Hornung G, Bar-Ziv R, Rosin D, Tokuriki N, Tawfik DS, Oren M, et al Noise-mean relationship in mutated promoters. Genome research. 2012;22: 2409–17. 10.1101/gr.139378.112 22820945 PMC3514670

[27] Choi JK, Kim Y-J. Intrinsic variability of gene expression encoded in nucleosome positioning sequences. Nature Genetics. 2009;41: 498–503. 10.1038/ng.319 19252489

[28] Martínez-Pastor MT, Marchler G, Schüller C, Marchler-Bauer A, Ruis H, Estruch F. The Saccharomyces cerevisiae zinc finger proteins Msn2p and Msn4p are required for transcriptional induction through the stress response element (STRE). The EMBO Journal. 1996;15: 2227–2235. 10.1002/j.1460-2075.1996.tb00576.x 8641288 PMC450147

[29] Bar-Even A, Paulsson J, Maheshri N, Carmi M, O’Shea E, Pilpel Y, et al Noise in protein expression scales with natural protein abundance. Nature Genetics. 2006;38: 636–643. 10.1038/ng1807 16715097

[30] Raj A, van den Bogaard P, Rifkin SA, van Oudenaarden A, Tyagi S. Imaging individual mRNA molecules using multiple singly labeled probes. Nature Methods. 2008;5: 877–879. 10.1038/nmeth.1253 18806792 PMC3126653

[31] Munsky B, Neuert G, van Oudenaarden A. Using gene expression noise to understand gene regulation. Science (New York, NY). 2012;336: 183–7. 10.1126/science.1216379 22499939 PMC3358231

[32] Keren L, Hausser J, Lotan-Pompan M, Vainberg Slutskin I, Alisar H, Kaminski S, et al Massively Parallel Interrogation of the Effects of Gene Expression Levels on Fitness. Cell. 2016;166: 1282–1294.e18. 10.1016/j.cell.2016.07.024 27545349

[33] Fraser HB, Hirsh AE, Giaever G, Kumm J, Eisen MB. Noise Minimization in Eukaryotic Gene Expression. Ken Wolfe, editor. PLoS Biology. 2004;2: e137 10.1371/journal.pbio.0020137 15124029 PMC400249

[34] Wang Z, Zhang J. Impact of gene expression noise on organismal fitness and the efficacy of natural selection. Proceedings of the National Academy of Sciences of the United States of America. 2011;108: E67–76. 10.1073/pnas.1100059108 21464323 PMC3080991

[35] Richard M, Yvert G. How does evolution tune biological noise? Frontiers in Genetics. 2014;5: 374 10.3389/fgene.2014.00374 25389435 PMC4211553

[36] Huh W-K, Falvo J V., Gerke LC, Carroll AS, Howson RW, Weissman JS, et al Global analysis of protein localization in budding yeast. Nature. 2003;425: 686–691. 10.1038/nature02026 14562095

[37] Breker M, Gymrek M, Moldavski O, Schuldiner M. LoQAtE—Localization and Quantitation ATlas of the yeast proteomE. A new tool for multiparametric dissection of single-protein behavior in response to biological perturbations in yeast. Nucleic Acids Research. 2014;42: D726–D730. 10.1093/nar/gkt933 24150937 PMC3965041

[38] Petrenko N, Chereji R V., McClean MN, Morozov A V., Broach JR. Noise and interlocking signaling pathways promote distinct transcription factor dynamics in response to different stresses. Edelstein-Keshet L, editor. Molecular Biology of the Cell. 2013;24: 2045–2057. 10.1091/mbc.E12-12-0870 23615444 PMC3681706

[39] Hao N, Budnik BA, Gunawardena J, O’Shea EK. Tunable signal processing through modular control of transcription factor translocation. Science (New York, NY). 2013;339: 460–4. 10.1126/science.1227299 23349292 PMC3746486

[40] Lin Y, Sohn CH, Dalal CK, Cai L, Elowitz MB. Combinatorial gene regulation by modulation of relative pulse timing. Nature. 2015;527: 54–58. 10.1038/nature15710 26466562 PMC4870307

[41] AkhavanAghdam Z, Sinha J, Tabbaa OP, Hao N. Dynamic control of gene regulatory logic by seemingly redundant transcription factors. eLife. 2016;5: e18458 10.7554/eLife.18458 27690227 PMC5047750

[42] Zentner GE, Kasinathan S, Xin B, Rohs R, Henikoff S. ChEC-seq kinetics discriminates transcription factor binding sites by DNA sequence and shape in vivo. Nature Communications. 2015;6: 8733 10.1038/ncomms9733 26490019 PMC4618392

[43] Siggers T, Reddy J, Barron B, Bulyk ML. Diversification of Transcription Factor Paralogs via Noncanonical Modularity in C2H2 Zinc Finger DNA Binding. Molecular Cell. 2014;55: 640–648. 10.1016/j.molcel.2014.06.019 25042805 PMC4142112

[44] Hibbs MA, Hess DC, Myers CL, Huttenhower C, Li K, Troyanskaya OG. Exploring the functional landscape of gene expression: directed search of large microarray compendia. Bioinformatics. 2007;23: 2692–2699. 10.1093/bioinformatics/btm403 17724061

[45] Kemmeren P, Sameith K, van de Pasch LAL, Benschop JJ, Lenstra TL, Margaritis T, et al Large-Scale Genetic Perturbations Reveal Regulatory Networks and an Abundance of Gene-Specific Repressors. Cell. 2014;157: 740–752. 10.1016/j.cell.2014.02.054 24766815

[46] Weiner A, Hsieh T-HS, Appleboim A, Chen HV, Rahat A, Amit I, et al High-Resolution Chromatin Dynamics during a Yeast Stress Response. Molecular Cell. 2015;58: 371–386. 10.1016/j.molcel.2015.02.002 25801168 PMC4405355

[47] Park D, Morris AR, Battenhouse A, Iyer VR. Simultaneous mapping of transcript ends at single-nucleotide resolution and identification of widespread promoter-associated non-coding RNA governed by TATA elements. Nucleic Acids Research. 2014;42: 3736–3749. 10.1093/nar/gkt1366 24413663 PMC3973313

[48] MacIsaac KD, Wang T, Gordon DB, Gifford DK, Stormo GD, Fraenkel E. An improved map of conserved regulatory sites for Saccharomyces cerevisiae. BMC Bioinformatics. 2006;7: 113 10.1186/1471-2105-7-113 16522208 PMC1435934

[49] Spealman P, Naik AW, May GE, Kuersten S, Freeberg L, Murphy RF, et al Conserved non-AUG uORFs revealed by a novel regression analysis of ribosome profiling data. Genome research. 2018;28: 214–222. 10.1101/gr.221507.117 29254944 PMC5793785

[50] Thompson DA, Roy S, Chan M, Styczynski MP, Pfiffner J, French C, et al Evolutionary principles of modular gene regulation in yeasts. 2013;2: 603 10.7554/eLife.00603 23795289 PMC3687341

[51] Field Y, Kaplan N, Fondufe-Mittendorf Y, Moore IK, Sharon E, Lubling Y, et al Distinct Modes of Regulation by Chromatin Encoded through Nucleosome Positioning Signals. Ohler U, editor. PLoS Computational Biology. 2008;4: e1000216 10.1371/journal.pcbi.1000216 18989395 PMC2570626

[52] Tirosh I, Barkai N. Two strategies for gene regulation by promoter nucleosomes. Genome Research. 2008;18: 1084–1091. 10.1101/gr.076059.108 18448704 PMC2493397

[53] Nicolas D, Phillips NE, Naef F. What shapes eukaryotic transcriptional bursting? Molecular BioSystems. 2017;13: 1280–1290. 10.1039/c7mb00154a 28573295

[54] Kuang Z, Pinglay S, Ji H, Boeke JD. Msn2/4 regulate expression of glycolytic enzymes and control transition from quiescence to growth. Elife. 2017;6: e29938 10.7554/eLife.29938 28949295 PMC5634782

[55] Tirosh I, Wong KH, Barkai N, Struhl K. Extensive divergence of yeast stress responses through transitions between induced and constitutive activation. Proceedings of the National Academy of Sciences of the United States of America. 2011;108: 16693–8. 10.1073/pnas.1113718108 21930916 PMC3189053

[56] Kafri R, Springer M, Pilpel Y. Genetic Redundancy: New Tricks for Old Genes. Cell. 2009;136: 389–392. 10.1016/j.cell.2009.01.027 19203571

[57] Alon U. Network motifs: theory and experimental approaches. Nature Reviews Genetics. 2007;8: 450–461. 10.1038/nrg2102 17510665

[58] Teichmann SA, Babu MM. Gene regulatory network growth by duplication. Nature Genetics. 2004;36: 492–496. 10.1038/ng1340 15107850

[59] Huber F, Meurer M, Bunina D, Kats I, Maeder CI, Štefl M, et al PCR Duplication: A One-Step Cloning-Free Method to Generate Duplicated Chromosomal Loci and Interference-Free Expression Reporters in Yeast. Schacherer J, editor. PLoS ONE. 2014;9: e114590 10.1371/journal.pone.0114590 25493941 PMC4262419

[60] Rahman S, Zenklusen D. Single-molecule resolution fluorescent in situ hybridization (smFISH) in the yeast S. cerevisiae. Methods in Molecular Biology. 2013;1042: 33–46. 10.1007/978-1-62703-526-2_3 23979998

[61] Avraham N, Soifer I, Carmi M, Barkai N. Increasing population growth by asymmetric segregation of a limiting resource during cell division. Molecular Systems Biology. 2013;9: 656 10.1038/msb.2013.13 23591772 PMC3658268

[62] Kafri M, Metzl-Raz E, Jona G, Barkai N. The Cost of Protein Production. Cell Reports. 2016;14: 22–31. 10.1016/j.celrep.2015.12.015 26725116 PMC4709330

[63] Orsi GA, Kasinathan S, Zentner GE, Henikoff S, Ahmad K. Mapping Regulatory Factors by Immunoprecipitation from Native Chromatin. Curr Protoc Mol Biol. 2015;110: 21.31.1–25. 10.1002/0471142727.mb2131s110 25827087 PMC4410783

[64] Pelechano V, Wei W, Steinmetz LM. Extensive transcriptional heterogeneity revealed by isoform profiling. Nature. 2013;497: 127–131. 10.1038/nature12121 23615609 PMC3705217

[65] De Boer CG, Hughes TR. YeTFaSCo: A database of evaluated yeast transcription factor sequence specificities. Nucleic Acids Research. 2012;40: D169–D179. 10.1093/nar/gkr993 22102575 PMC3245003

[66] Gupta S, Stamatoyannopoulos JA, Bailey TL, Noble W. Quantifying similarity between motifs. Genome Biology. 2007;8: R24 10.1186/gb-2007-8-2-r24 17324271 PMC1852410

[67] Ihmels J, Bergmann S, Barkai N. Defining transcription modules using large-scale gene expression data. Bioinformatics. 2004;20: 1993–2003. 10.1093/bioinformatics/bth166 15044247

