# Supplementary figures and images for: Resolving noise–control conflict by gene duplication
Source: PLoS Biol. 2019 Nov 22;17(11):e3000289. doi: 10.1371/journal.pbio.3000289 (PMC6874299; doi:10.1371/journal.pbio.3000289)

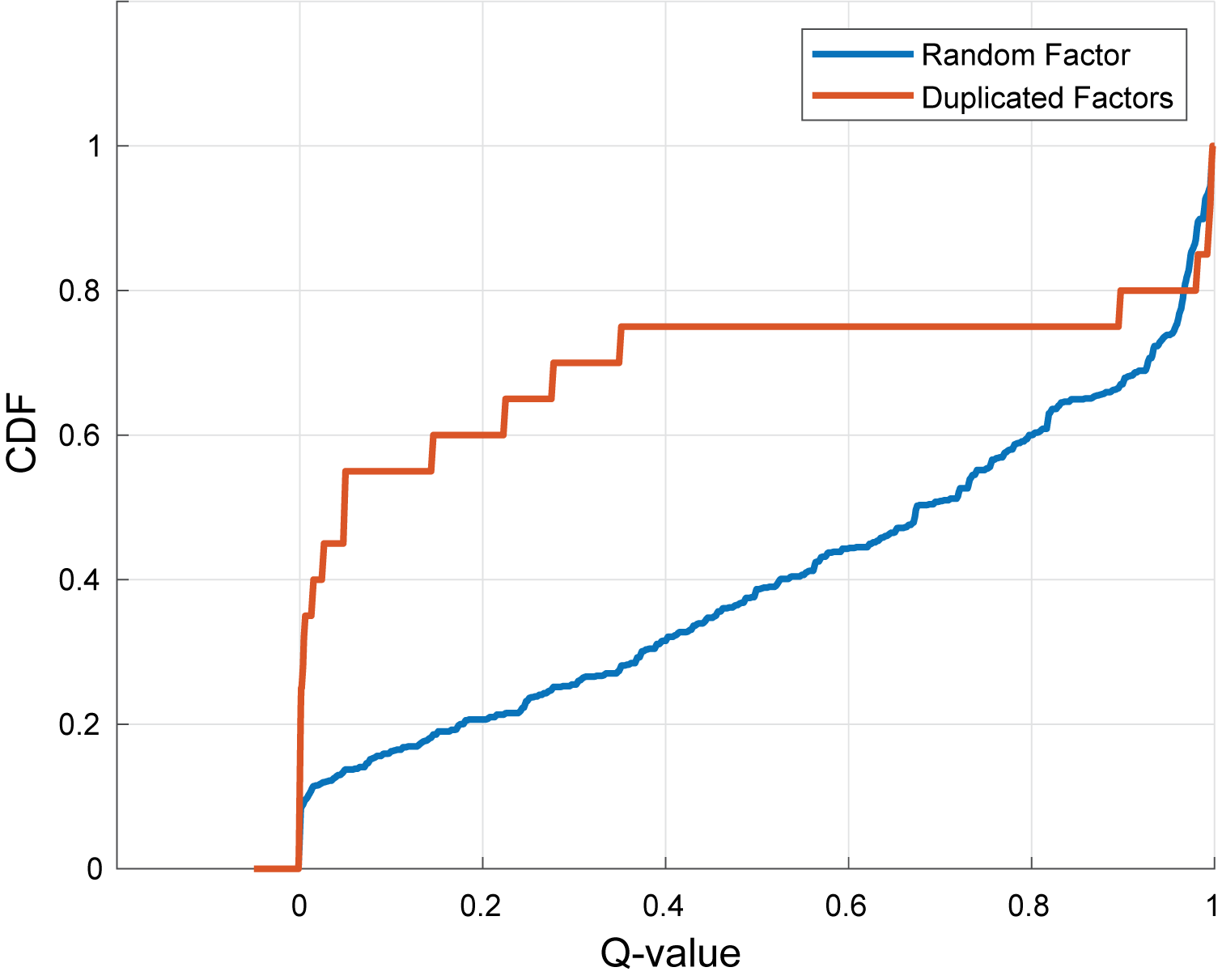

Supplement: S1 Fig — We used available position frequency matrices of all available DNA-binding motifs in YeTFaSCo [65] (“expert collection”) and measured similarity using Tomtom [66]. Here, we show the CDFs of the Q-values similarities between motifs of duplicated TFs (red) and random TFs (blue). CDF, cumulative distribution function; TF, transcription factor; YeTFaSCo, Yeast Transcription Factor Specificity Compendium. (TIF) [file pbio.3000289.s001.tif]

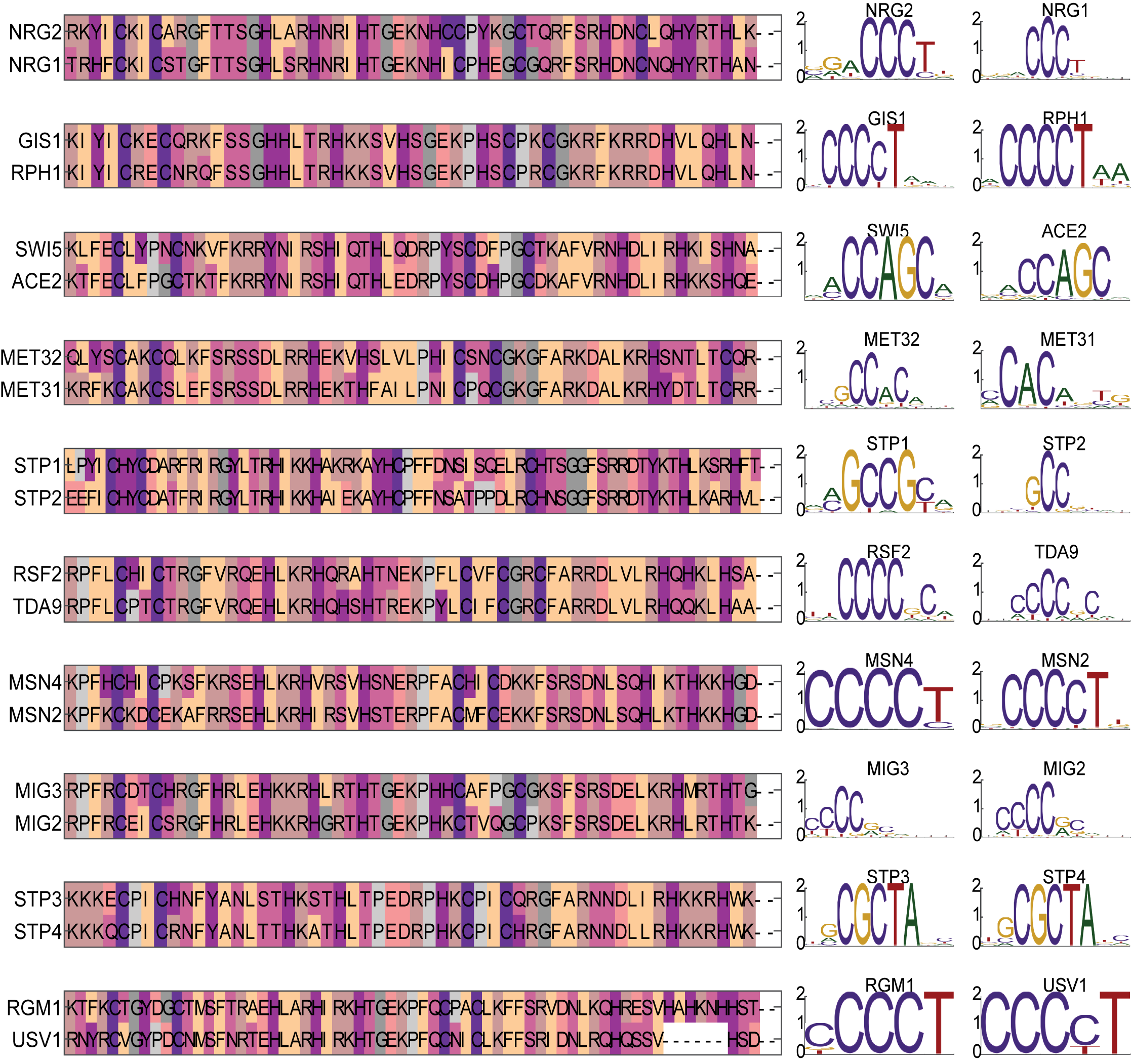

Supplement: S2 Fig — (Left) Alignment of binding domains of all duplicated pairs. (Right) DNA-binding motifs of the pairs from YeTFaSCo [65]. TF, transcription factor; WGD, Whole Genome Duplication; YeTFaSCo, Yeast Transcription Factor Specificity Compendium. (TIF) [file pbio.3000289.s002.tif]

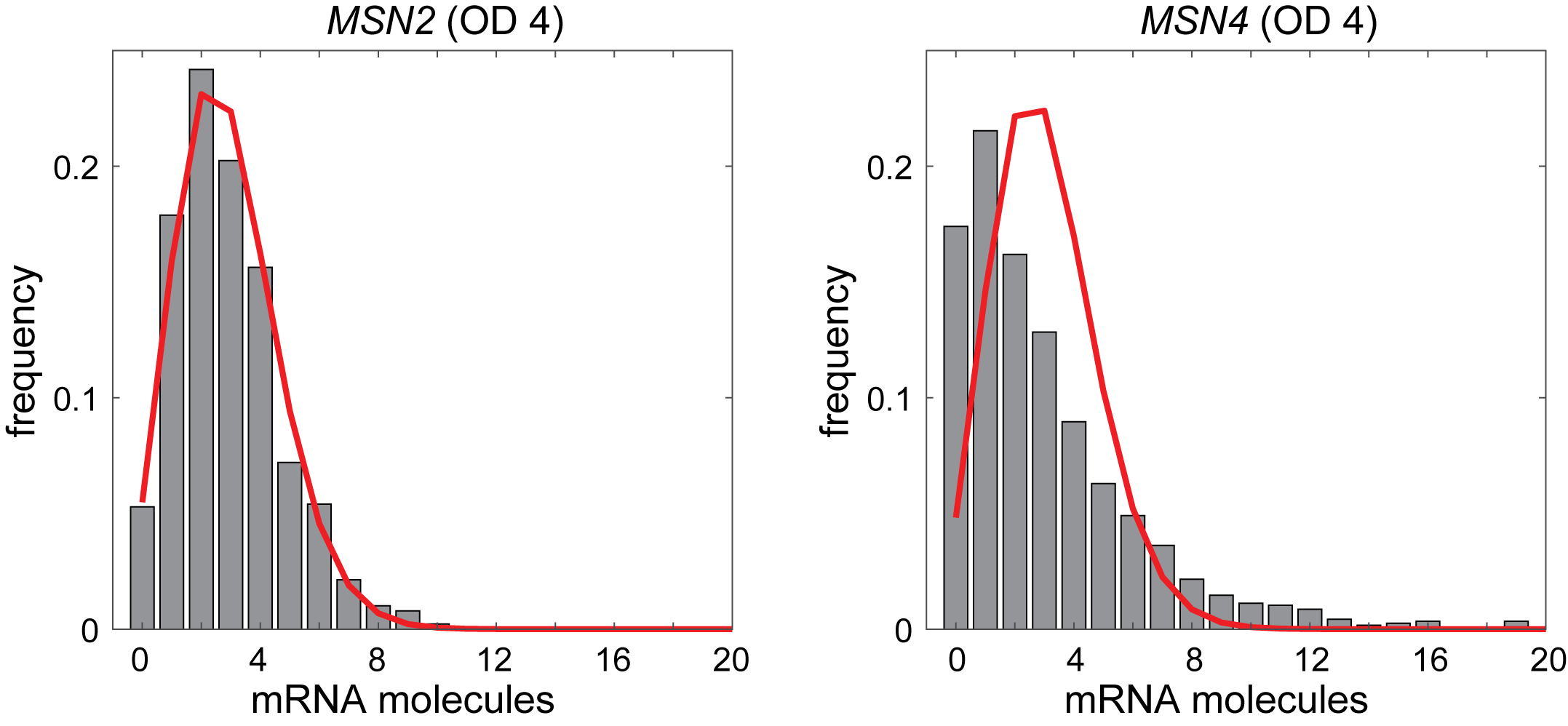

Supplement: S3 Fig — MSN2 (left) and MSN4 (right) expression levels were measured by smFISH at OD600 4, where both TFs showed similar mean expression. Shown are mRNA molecule count distributions. Red lines represent the best Poisson fit to the data. Raw data are available in S1 Data. OD, Optical Density; smFISH, single-molecule Fluorescent In Situ Hybridization; TF, transcription factor. (TIF) [file pbio.3000289.s003.tif]

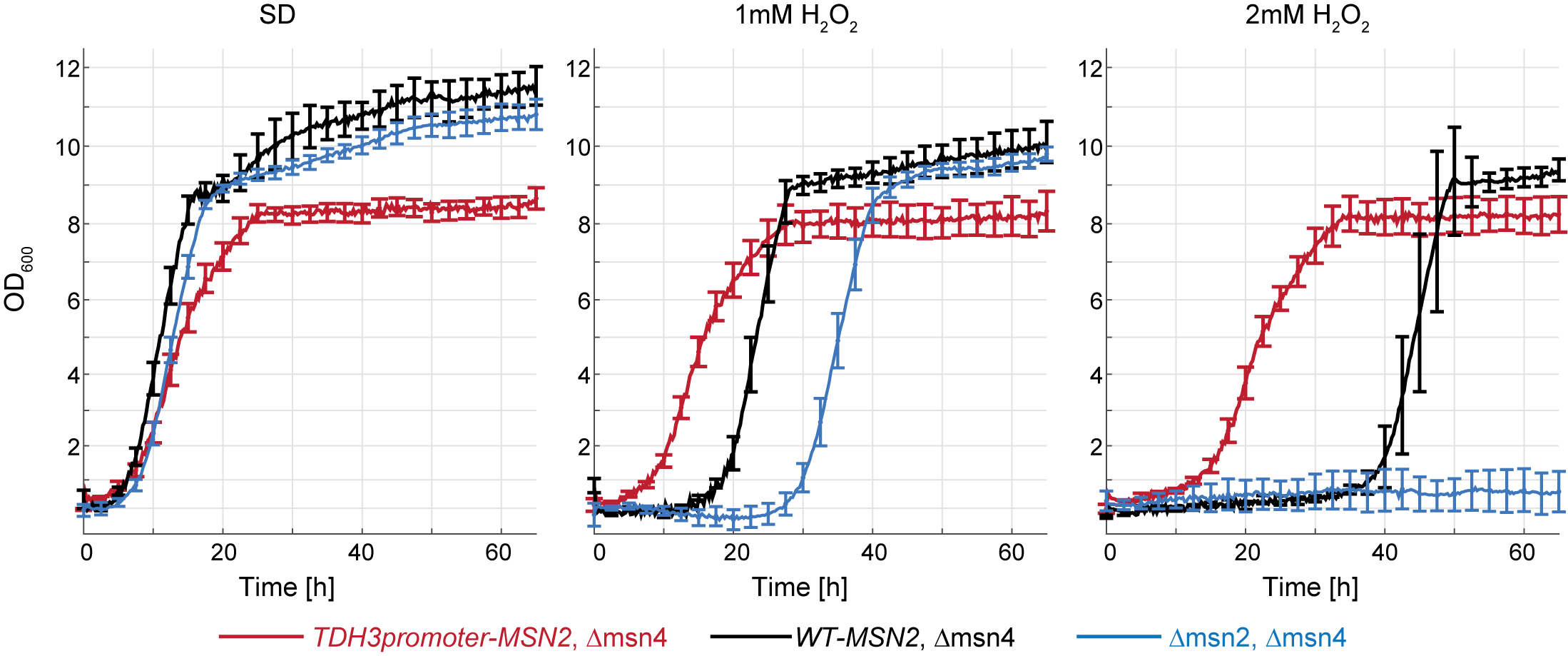

Supplement: S4 Fig — Cells were grown in the indicated condition under constant shaking and 30°C in 96-well plates in an automated handling robot (EVOware, Tecan Inc.). OD was measured automatically approximately every 30 minutes for 65 hours using Infinite200 reader. Raw data are available in S3 Data. OD, Optical Density; SC, synthetic complete. (TIF) [file pbio.3000289.s004.tif]

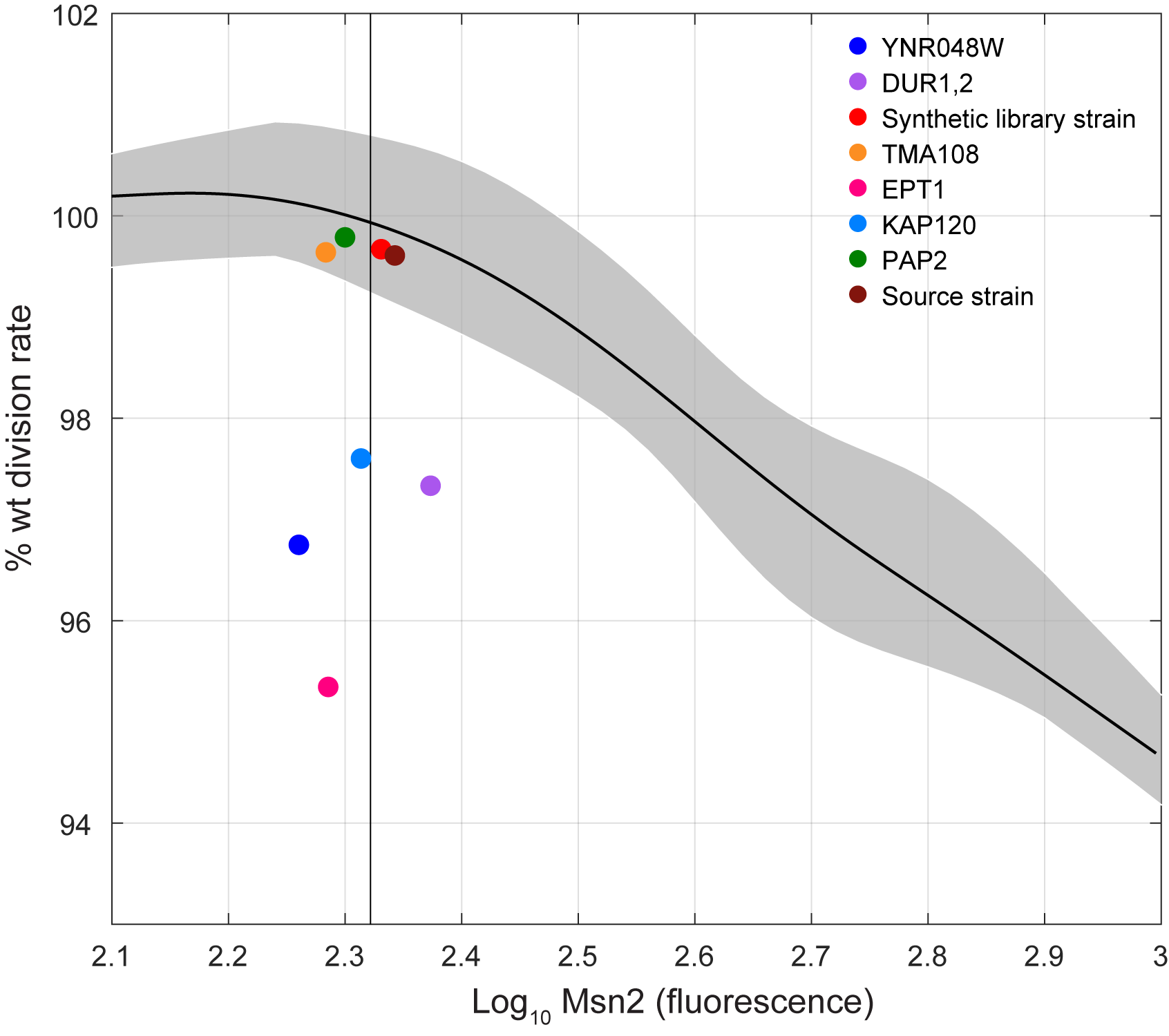

Supplement: S5 Fig — Noisy Msn2 strains were generated by swapping the endogenous MSN2 promoter (“source strain”; dark red), with other, noisier gene promoters. Shown are these strains and one strain from the synthetic library strain, as indicated in the legend. Gray shade indicates the synthetic library strains phenotype for a reference (see Fig 1D for details). Raw data are available in S2 Data. GFP, green fluorescent protein. (TIF) [file pbio.3000289.s005.tif]

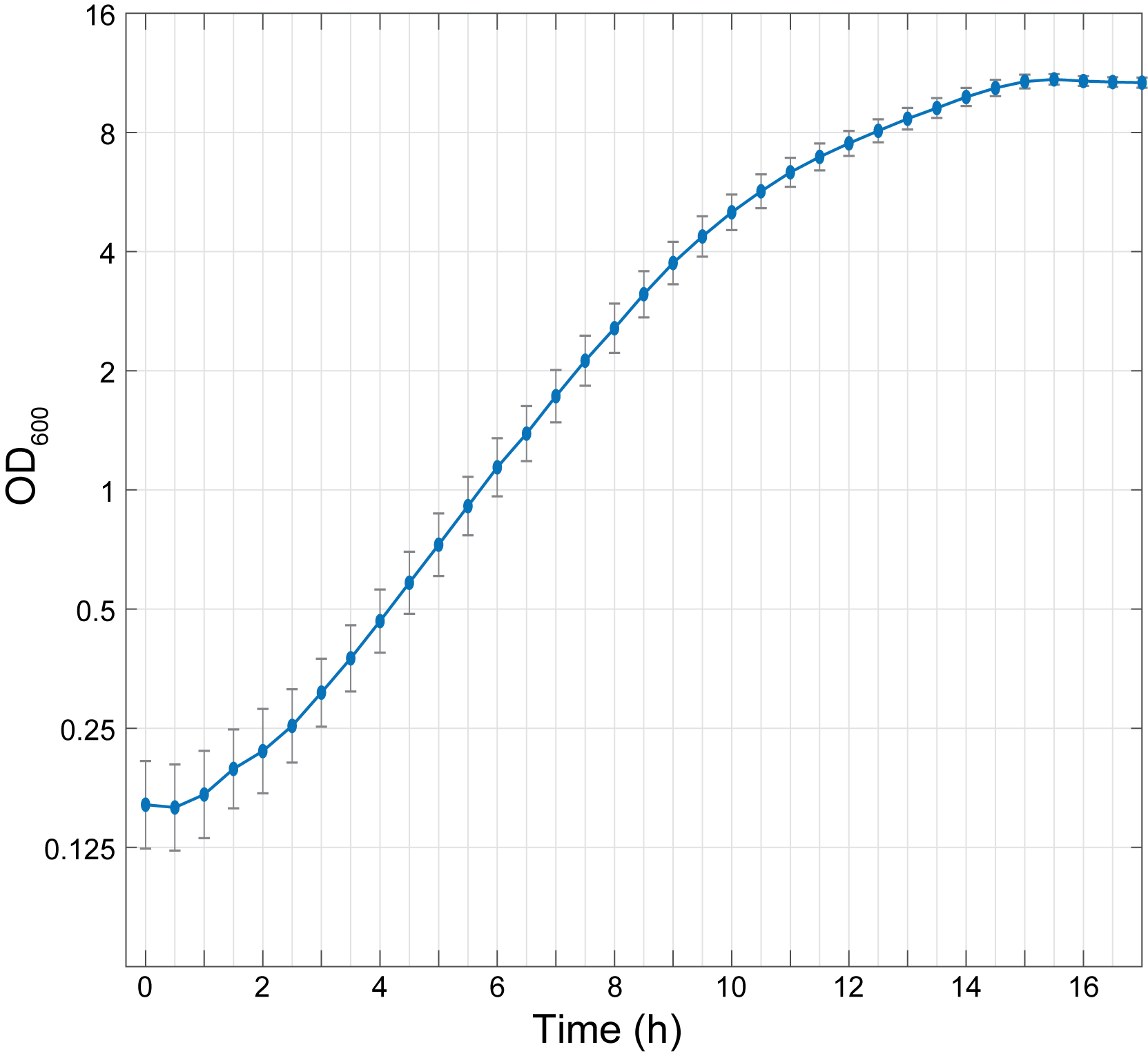

Supplement: S6 Fig — Shown are OD measurements on the y-axis (logarithmic scale) as a function of time. Error bars represent standard deviation of 16 repeats. OD, Optical Density. (TIF) [file pbio.3000289.s006.tif]

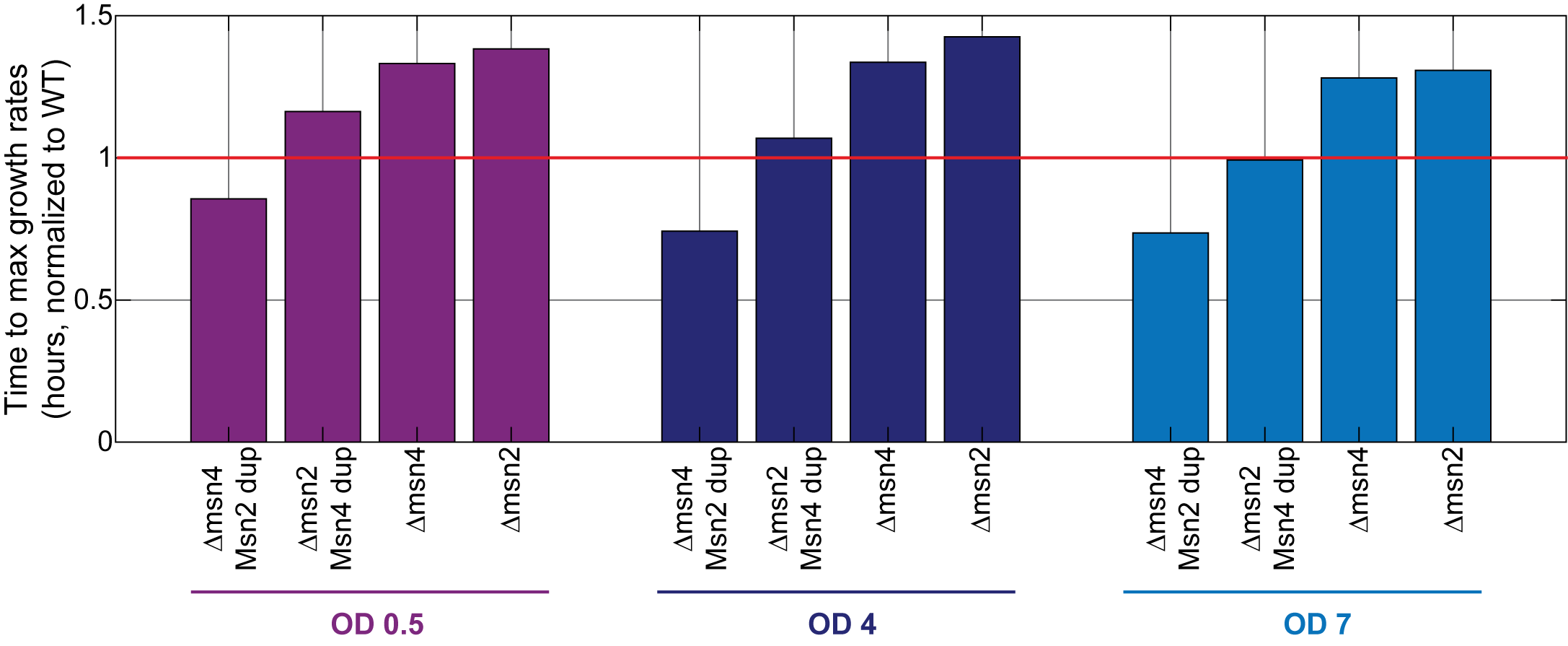

Supplement: S7 Fig — We generated a strain with MSN2 duplication and msn4 deletion and a strain with MSN4 duplication and MSN2 deletion. We measured stress protection by diluting cells at different stages along the growth curve into media containing 1.6 mM H2O2 and measuring OD continuously to define the time at which maximal growth was first detected. Shown is the time to resume maximal growth, normalized to the time it took the WT strain to resume maximal growth. Red line represents the WT strain. Raw data are available in S3 Data. OD, Optical Density; WT, wild type. (TIF) [file pbio.3000289.s007.tif]

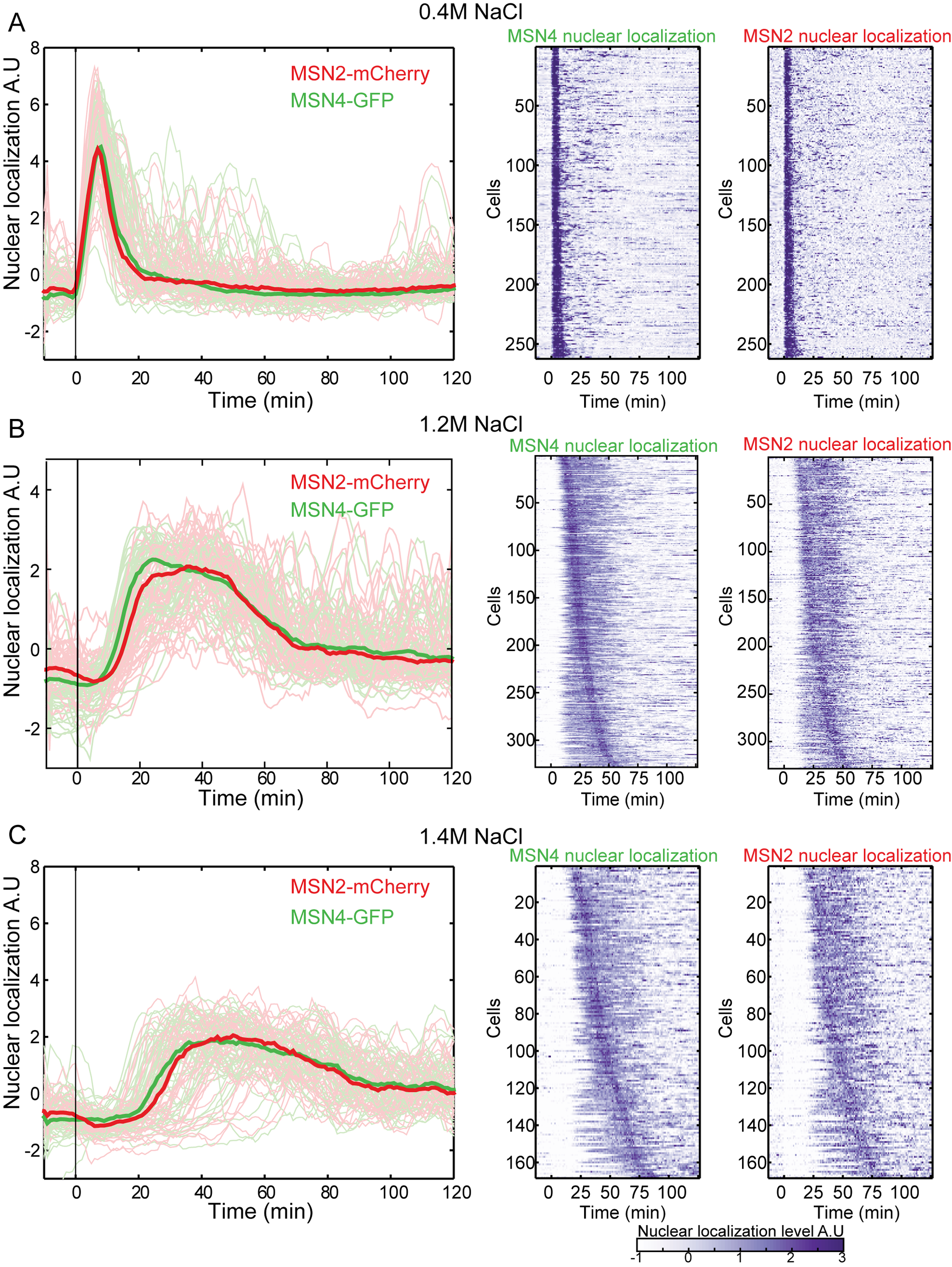

Supplement: S8 Fig — Single cells expressing Msn4-GFP and Msn2-mCherry fusion proteins were tracked using microfluidics-coupled live microscopy in 0.4/1.2/1.4 M NaCl. (Left) Localization dynamics following exposure to stress is shown as the medians, and the single cell traces are shown as shaded lines. (Right) Individual nuclear localization traces of both Msn2 and Msn4 are shown, with cells in both columns presented in the same order. Raw data are available in S5 Data. GFP, green fluorescent protein; Msn, XXX. (TIF) [file pbio.3000289.s008.tif]

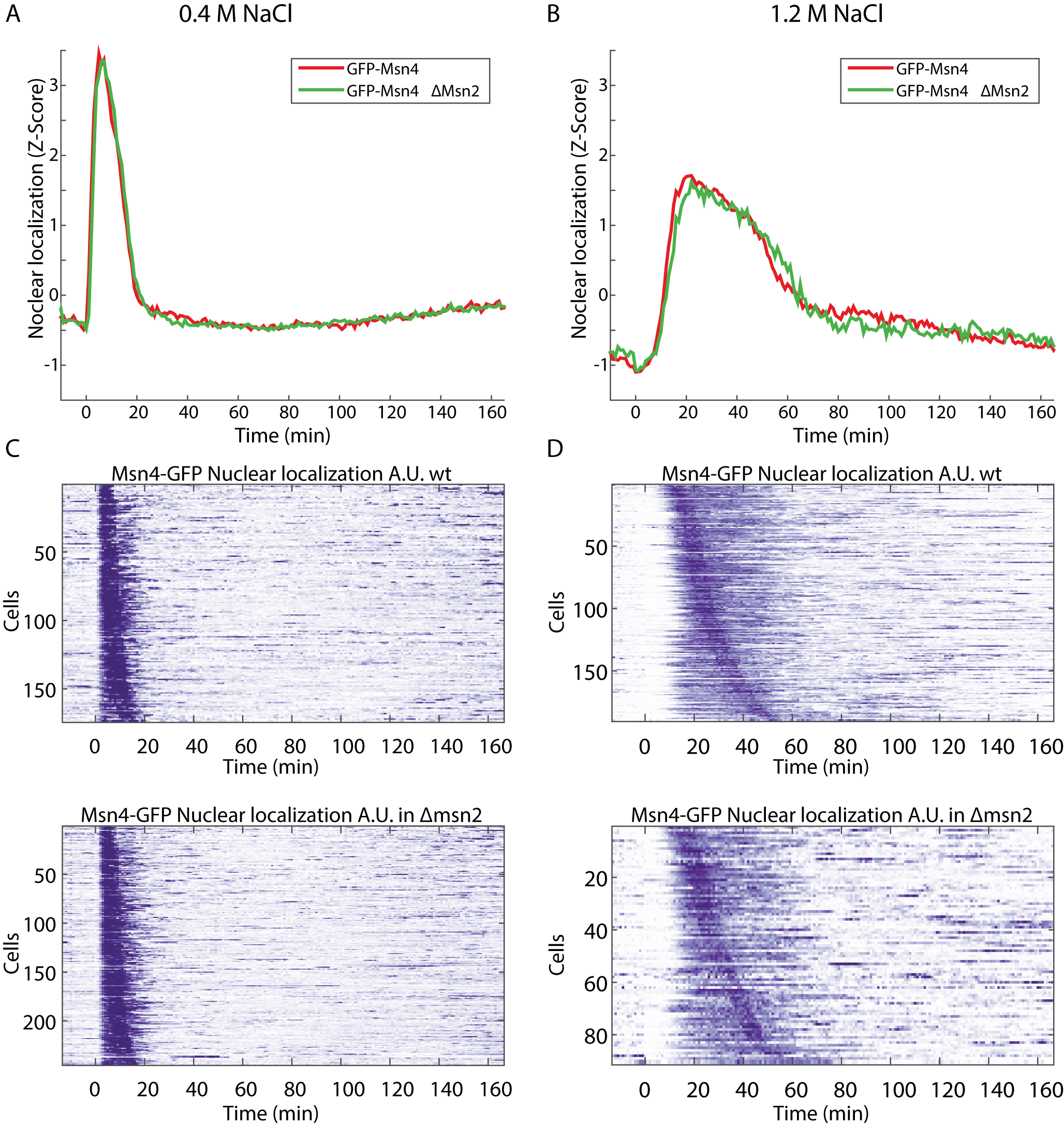

Supplement: S9 Fig — (A,B) Localization dynamics following exposure to 0.4/1.2M NaCl is shown as the median. (C,D) Individual traces of Msn4 in WT cells of cells deleted of msn2. Raw data are available in S5 Data. WT, wild type. (TIF) [file pbio.3000289.s009.tif]

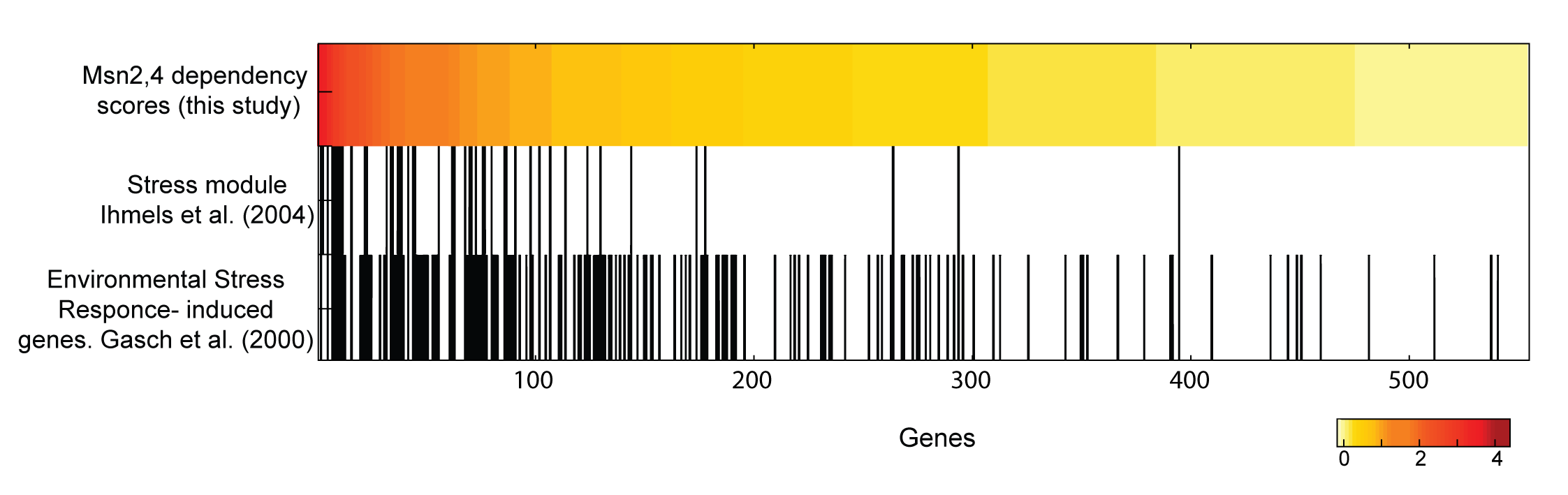

Supplement: S10 Fig — We calculated Msn2,4 dependency score for each gene as the average over all conditions, of ratio between WT induction and the double msn2, msn4 deletion strain induction. The 500 top Msn2,4-dependent genes are ordered by this score. Shown are the scores and an indication if the genes are part of the written published data sets [13,67] (black: gene is part of the group, white: gene is not part of the group). Raw data are available at SRA under BioProject PRJNA541833. SRA, Sequence Read Archive; WT, wild type. (TIF) [file pbio.3000289.s010.tif]

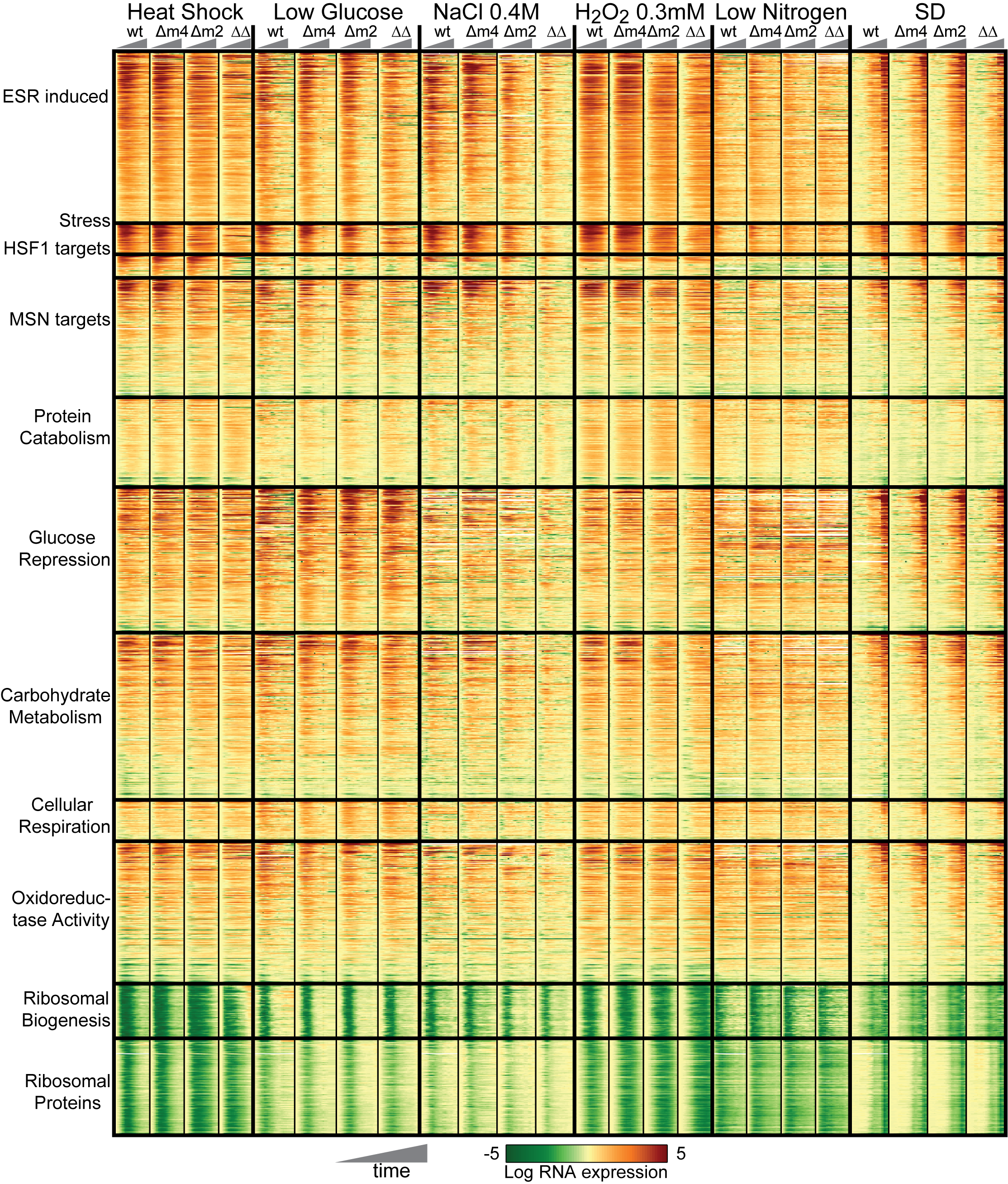

Supplement: S11 Fig — Cells were grown to exponential phase; then, at OD600 0.2–0.4, they were exposed to stress. Samples for mRNA measurements were taken every 3 minutes for the first hour after stress induction and every 10 minutes for the next one/half an hour. In addition, we took samples along the growth curve every 20–30 minutes (SC). Raw data are available at SRA under BioProject PRJNA541833. OD, Optical Density; SC, synthetic complete; SRA, Sequence Read Archive. (TIF) [file pbio.3000289.s011.tif]

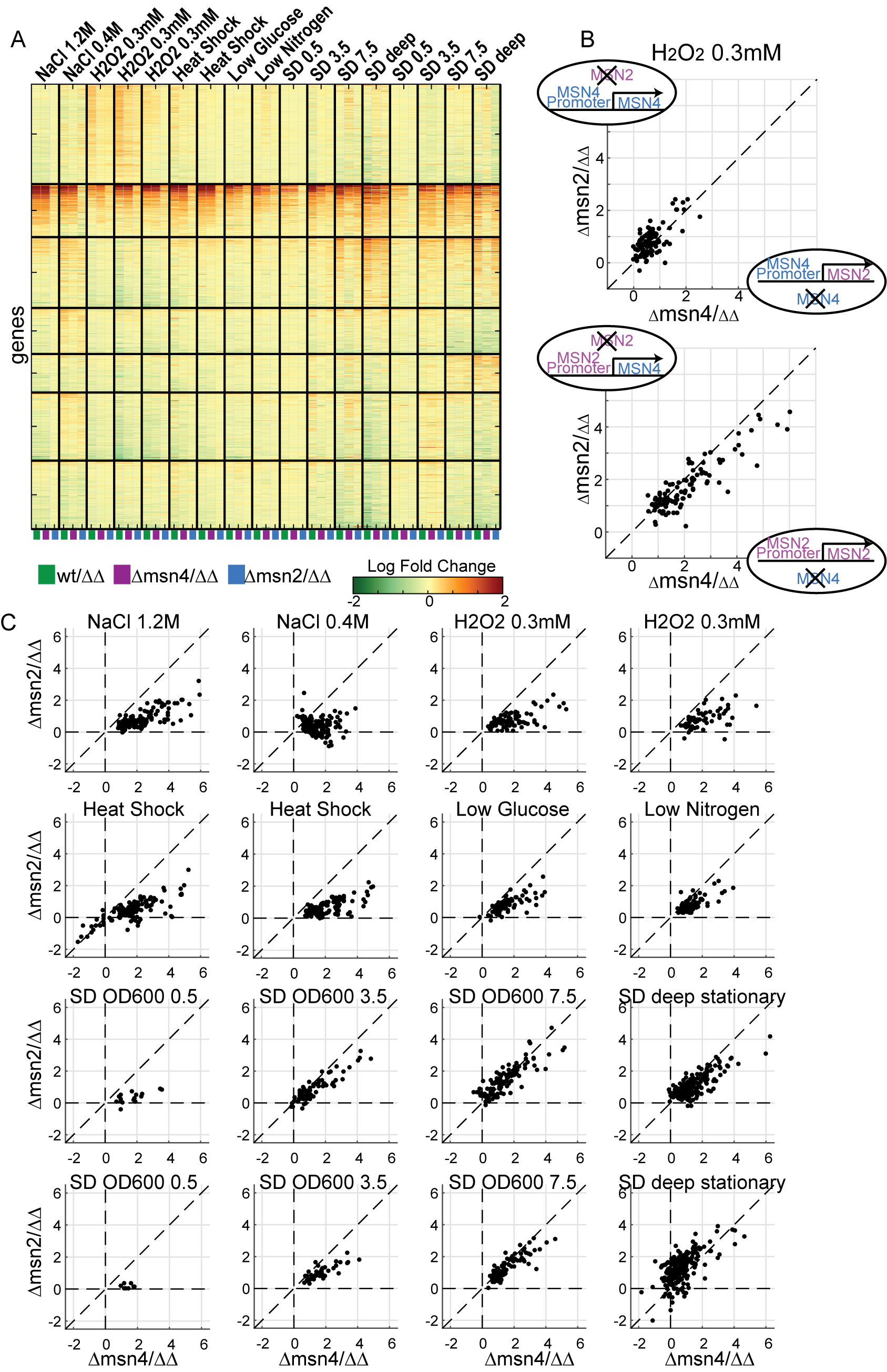

Supplement: S12 Fig — (A) Clustering of all genes in all the conditions and repeats that we checked. For each experiment of the stress perturbations, we calculated for each strain the AUC, and for cells growing into the stationary phase, we used expression in different ODs. We then calculated the fold change of WT or single deletions to the double-deletion strain and used these values to cluster genes. (B) Swapping Msn2,4 promoter. Shown is the fold change of gene induction in response to H2O2 in the indicated strains relative to the double-deletion strain. Each dot represents a gene that was >2-fold higher in the WT then the double deletion. (C) Shown is the fold change of gene-induction different stress conditions in the single-deletion strains relative to the double-deletion strain. Each dot represents a gene that was >2-fold higher in the WT then the double-deletion strain. Raw data are available at SRA under BioProject PRJNA541833. AUC, area under the curve; OD, Optical Density; SRA, Sequence Read Archive; WT, wild type. (TIF) [file pbio.3000289.s012.tif]

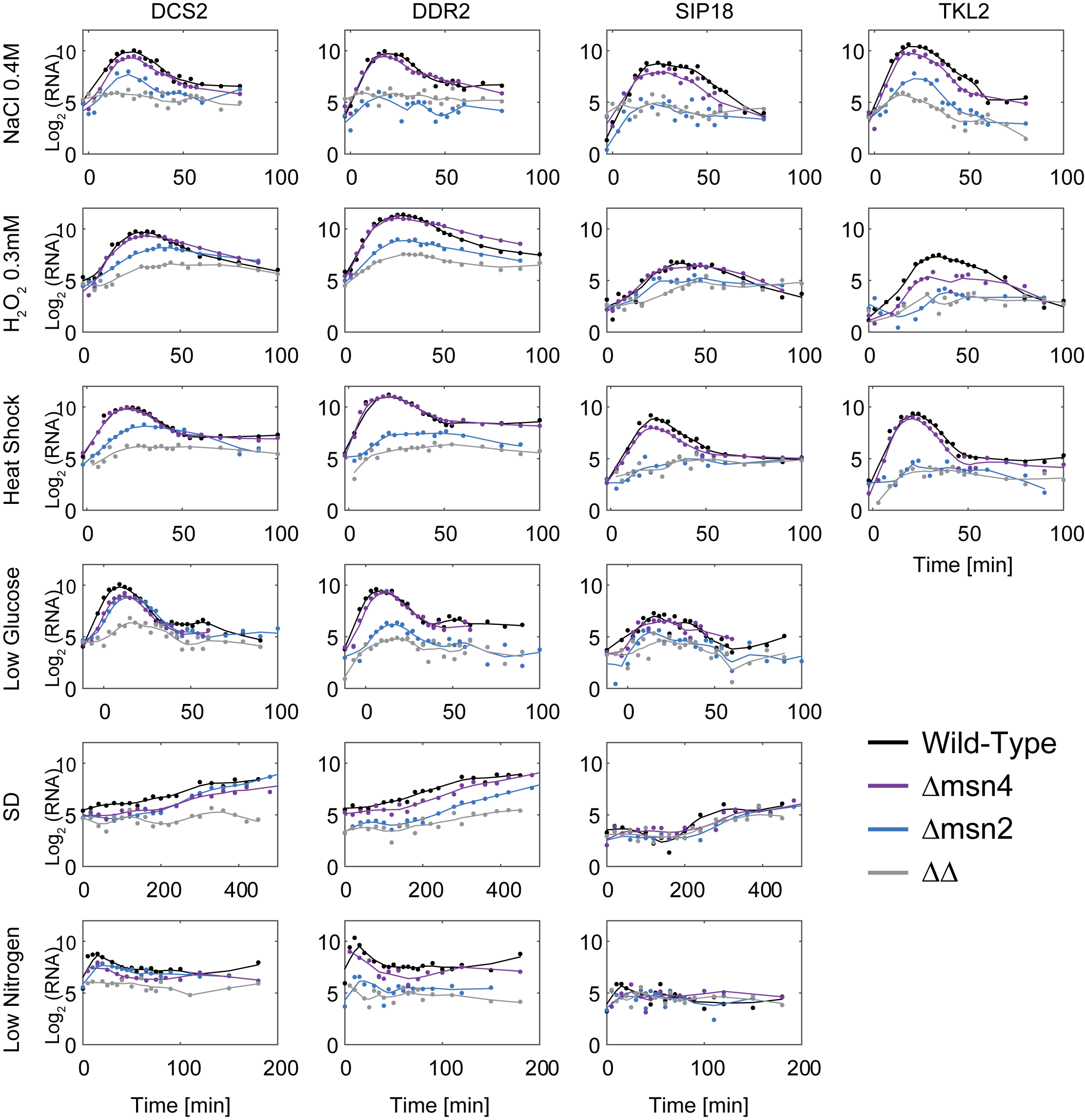

Supplement: S13 Fig — Plotted are mRNA measurements (from our study) of the response of the four genes reported in AkhavanAghdam and colleagues [41]. Shown are mRNA measurements for the WT, single-, and double-deletion msn2,4 strains in response to various stress conditions. All stresses were introduced to cells growing exponentially (0.2–0.4 OD600). In addition, we measured mRNA expression along the growth curve (SD). Dots represent the data measurements, and lines are the smoothed signal. In our high-temporal–resolution data, there is no fundamental difference in Msn2,4 contribution to the response between the first two genes (DSC2, DDR2) and last two genes (SIP18, TKL2) as was reported. In all of these genes, MSN4-deleted strains show similar expression and dynamics to the WT strain, but MSN2-deleted strains reduce the induction significantly. Raw data are available at SRA under BioProject PRJNA541833. OD, Optical Density; SRA, Sequence Read Archive; WT, wild type. (TIF) [file pbio.3000289.s013.tif]

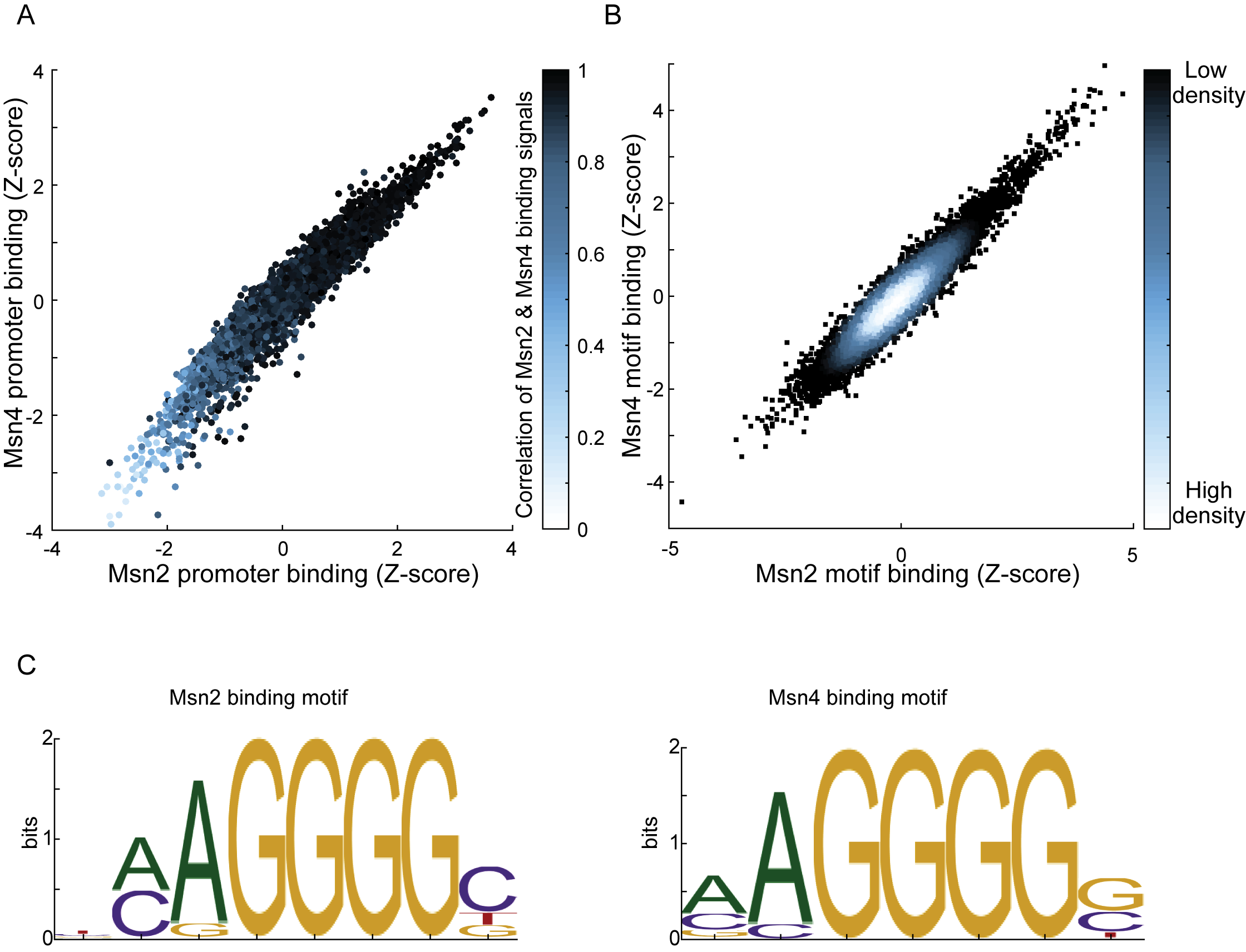

Supplement: S14 Fig — (A) Msn2 and Msn4 binding to all the promotors. Sum of the normalized ChEC-seq signal of each factor measured in cells at OD approximately 4 was calculated for all the promotes in >4 repeats. Shown is the z-score of the median of all repeats. Color represents the correlation of Msn2 and Msn4 binding signal on the promoters. (B) Density plot comparing Msn2 and Msn4 in vitro binding to all possible (8,192) 7-DNA base pair sequences. For each 7-mer, the mean signal of all its appearances in all promoters was calculated for Msn2 and Msn4. Shown is the density plot of the z-scores of all possible 7-mers. (C) DNA motifs found in our data for Msn2 and MSN4. Raw data are available at SRA under BioProject PRJNA573518. ChEC-seq, Chromatin Endogenous Cleavage sequencing; OD, Optical Density; SRA, Sequence Read Archive. (TIF) [file pbio.3000289.s014.tif]

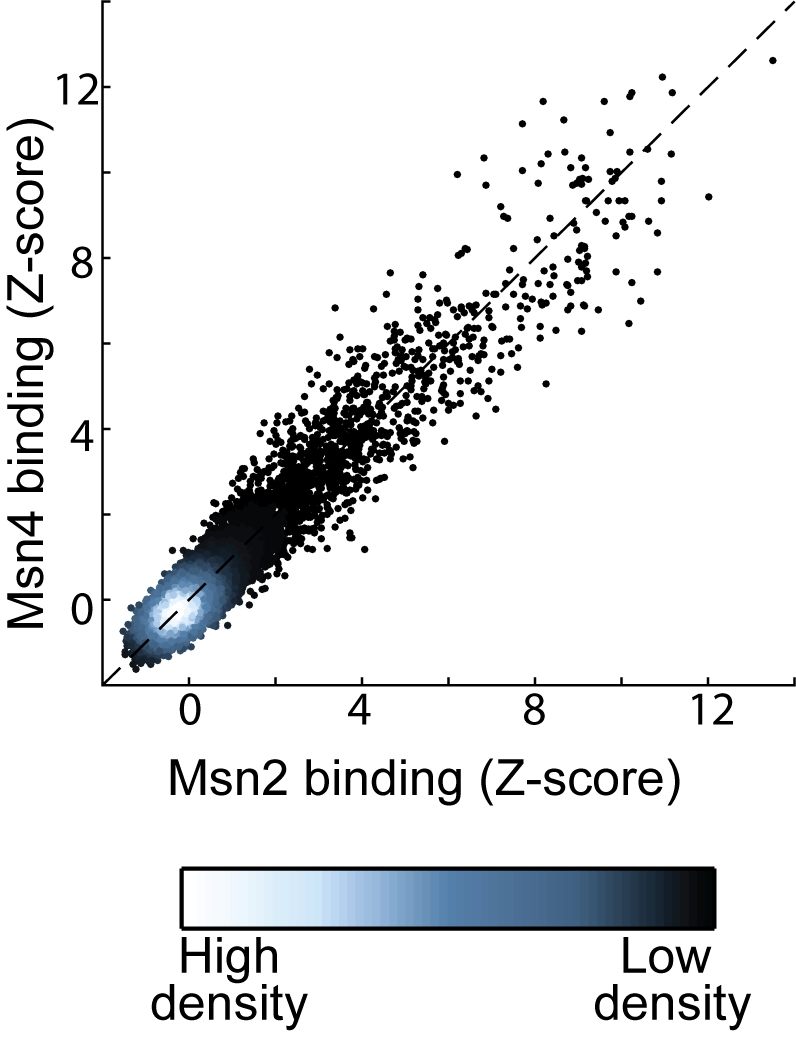

Supplement: S15 Fig — Density plot comparing Msn2,4 in vitro binding to all possible (32,896) 8-DNA base pair sequences. Data from Siggers and colleagues [43]. (TIF) [file pbio.3000289.s015.tif]

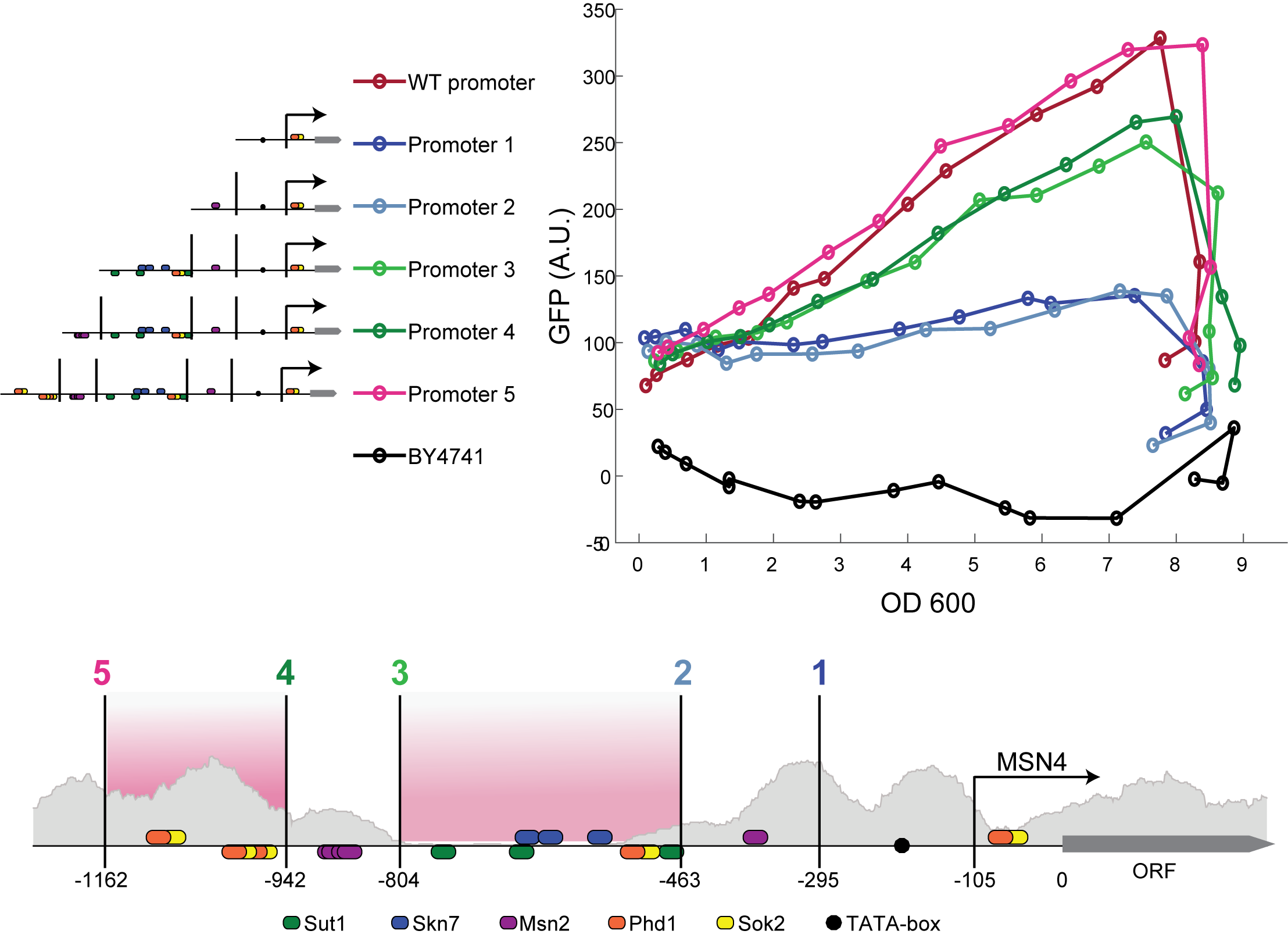

Supplement: S16 Fig — We generated five strains with partial MSN4 promoter by cutting the upstream part of the promoter in the indicated places in the scheme. Shown are median expression levels of Msn4-GFP along the growth curve in the strains with full and partial MSN4 promoter. The highlighted areas in the scheme show the promoter regions that induce Msn4 at high ODs. Raw data are available in S4 Data. GFP, green fluorescent protein; OD, Optical Density. (TIF) [file pbio.3000289.s016.tif]

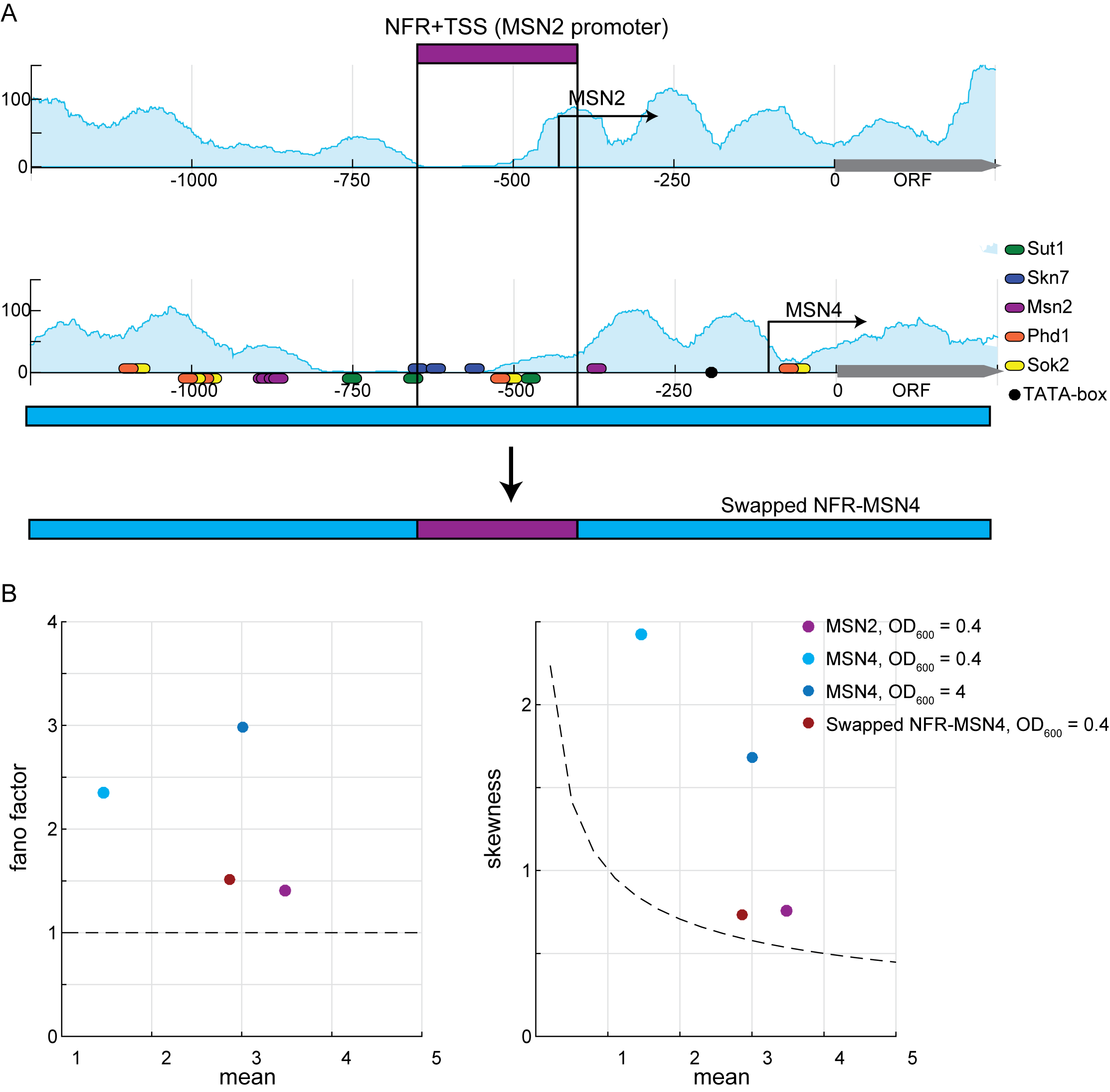

Supplement: S17 Fig — (A) A scheme of the strain we used—MSN4 promoter with a swap with MSN2 NFR+TSS in the same position. (B) smFISH results of the swapped strain and the WT MSN2,4 in the indicated ODs. Raw data are available in S1 Data. NFR, Nucleosome-Free Region; OD, Optical Density; smFISH, single-molecule Fluorescent In Situ Hybridization; TSS, Transcription Start Site; WT, wild type. (TIF) [file pbio.3000289.s017.tif]
