# Supplementary material for: Resolving noise–control conflict by gene duplication
Source: PLoS Biol. 2019 Nov 22;17(11):e3000289. doi: 10.1371/journal.pbio.3000289 (PMC6874299; doi:10.1371/journal.pbio.3000289)
Supplement: S1 Table — (DOCX) [file pbio.3000289.s023.docx]

S1 Table. Yeast strains used in this study.

|  | Strain | Genotype | Source |
| --- | --- | --- | --- |
| 1 | BY4741 | MATa his3Δ1 leu2Δ0 met15Δ0 ura3Δ0 | - |
| 2 | BY4742 | MATα his3Δ1 leu2Δ0 lys2Δ0 ura3Δ0 | - |
| 3 | wild-type | BY4741 | - |
| 4 | Δmsn2 | BY4741 msn2Δ:: hphNT1 | This study |
| 5 | Δmsn4 | BY4741 msn4Δ:: natNT2 | This study |
| 6 | Δmsn2Δmsn4 | BY4741 msn2Δ:: hphNT1 msn4Δ:: natNT2 | This study |
| 7 | Δmsn2, MSN4-promoter swap | BY4741 msn2Δ:: hphNT1 MSN4promoterΔ:: natNT2-MSN2promoter | This study |
| 8 | Δmsn4, MSN2-promoter swap | BY4741 msn4Δ:: natNT2 MSN2promoterΔ:: hphNT1-MSN4promoter | This study |
| 9 | Δmsn4, mCherry | BY4741 msn4Δ:: natNT2 HO::Kan+TDH3prom-mCherry | This study |
| 10 | Δmsn4 & MSN2-YFP | BY4741 msn4Δ:: natNT2 MSN2-YFP::kanMX4 | This study |
| 16 | Δmsn4 & MSN2 promoter library - 50 strains | BY4741 msn4Δ:: natNT2 MSN2-YFP::kanMX4 MSN2promoterΔ::URA-promoterX | This study |
| 17 | GFP-Msn4 | BY4742 GFP-MSN4 (N terminus tag), different colonies | This study, based on a strain from Yofe et al.[1] |
| 18 | GFP-Msn4 & Δmsn2 | BY4742 GFP-MSN4 msn2Δ:: hphNT1 | This study, based on strain 16 |
| 19 | GFP-Msn4 & Msn2-mCherry | BY4742 GFP-MSN4 MSN2- mCherry::His3 | This study, based on strain 16 |
| 20 | Msn2-GFP | BY4741 MSN2-GFP:: kanMX4 | This study |
| 21 | Msn2-Mnase | BY4741 MSN2-3FLAG-MNase:kanMX6 | This study |
| 22 | Msn4-Mnase | BY4741 MSN4-3FLAG-MNase:kanMX6 | This study |
| 23 | K.lactis promoter-Msn2 | BY4741 MSN2promoteΔ::K.lactisMSNpromoter | This study |
| 24 | MSN4 promoter with MSN2 promoter NFR+TSS | BY4741 MSN2promoter(400-650)Δ:: MSN4promoter(400-650) | This study |
| 25 | Δmsn4 & MSN2 promoter swapped by TMA108 promoter, MSN2-YFP | BY4741 msn4Δ:: natNT2 MSN2-YFP::HIS MSN2promoterΔ:: kanMX4-TMA108promoter | This study |
| 26 | Δmsn4 & MSN2 promoter swapped by KAP120 promoter, MSN2-YFP | BY4741 msn4Δ:: natNT2 MSN2-YFP::HIS MSN2promoterΔ:: KAP120promoter | This study |
| 27 | Δmsn4 & MSN2 promoter swapped by PAP2 promoter, MSN2-YFP | BY4741 msn4Δ:: natNT2 MSN2-YFP::HIS MSN2promoterΔ:: PAP2promoter | This study |
| 28 | Δmsn4 & MSN2 promoter swapped by YNR048W promoter, MSN2-YFP | BY4741 msn4Δ:: natNT2 MSN2-YFP::HIS MSN2promoterΔ:: YNR048Wpromoter | This study |
| 29 | Δmsn4 & MSN2 promoter swapped by EPT1 promoter, MSN2-YFP | BY4741 msn4Δ:: natNT2 MSN2-YFP::HIS MSN2promoterΔ:: EPT1promoter | This study |
| 30 | Δmsn4 & MSN2 promoter swapped by DUR1,2 promoter, MSN2-YFP | BY4741 msn4Δ:: natNT2 MSN2-YFP::HIS MSN2promoterΔ:: DUR1,2promoter | This study |
| 31 | Δmsn4 & MSN2 duplication | BY4741 msn4Δ:: natNT2 MSN2::MSN2- hphNT1-MSN2 | This study |
| 32 | Δmsn2 & MSN4 duplication | BY4741 msn2Δ:: hphNT1 MSN4::MSN4- natNT2-MSN4 | This study |
| 33 | Kluyveromyces lactis | CLIB 209 |  |
| 33 | Lachancea kluyveri | NRRL Y-12651 |  |

**References:**

1. Yofe I, Weill U, Meurer M, Chuartzman S, Zalckvar E, Goldman O, et al. One library to make them all: streamlining the creation of yeast libraries via a SWAp-Tag strategy. Nat Methods. 2016;13: 371–378. doi:10.1038/nmeth.3795
