# Supplementary material for: Resolving noise–control conflict by gene duplication
Source: PLoS Biol. 2019 Nov 22;17(11):e3000289. doi: 10.1371/journal.pbio.3000289 (PMC6874299; doi:10.1371/journal.pbio.3000289)
Supplement: S2 Table — smFISH, single-molecule Fluorescent In Situ Hybridization. (DOCX) [file pbio.3000289.s024.docx]

S2 Table. MSN2 smFISH probes, CAL Fluor Red 590

| PROBE # | PROBE (5'-> 3') |  | PROBE # | PROBE (5'-> 3') |  | PROBE # | PROBE (5'-> 3') |
| --- | --- | --- | --- | --- | --- | --- | --- |
| 1 | tgaaatcatggtcgaccgtc |  | 17 | agcatggagtctatgttcag |  | 33 | ccactttcgcaataacggac |
| 2 | ctactcatgctttctatggg |  | 18 | gctaaatcttcggcgtgata |  | 34 | tcattactcaagcctgtagt |
| 3 | ttgggttattattctccacg |  | 19 | tggttccaaaggtccaaaga |  | 35 | ttcaccttcctctgtcaaaa |
| 4 | ccgcactatctaagacagtg |  | 20 | cttgcattggaacttgaggt |  | 36 | ggagccatattcatttgagt |
| 5 | ccccaaattcagtgaagttt |  | 21 | ttgcgttcatagtagtggta |  | 37 | ttgcaagagacgtggaggat |
| 6 | gtagtggatggtatcgtttc |  | 22 | gttgccagcaatatttgagt |  | 38 | tccgaagggaagaaccgatt |
| 7 | tttgttgctatcagctttca |  | 23 | ctatggtagcgtcattgttt |  | 39 | tcaaaggcacagcagacttc |
| 8 | tcaatagttcttgcatcgcg |  | 24 | agtggatgtgcccaaattat |  | 40 | tgttgatttgtaagcggcac |
| 9 | gagccactagcattattagt |  | 25 | ggcaagcagatttgttcttg |  | 41 | gagttgacactactactgct |
| 10 | gagttgtgtattgatttgcc |  | 26 | atctttgtcatagcattggc |  | 42 | tgtcattgattttctcctgt |
| 11 | gtgacggtgattgtaacgga |  | 27 | tgctgtaattgctgacttgg |  | 43 | cgacacttgatcttctggac |
| 12 | ggattttgaggtaccgatga |  | 28 | tgtgttgaactcggtttagc |  | 44 | tcgagttcctttgttgattc |
| 13 | cttgctgtatttatgggagg |  | 29 | ttatgtgacgaggtgagctg |  | 45 | tgtgacagtggaacggtttc |
| 14 | ccatttgaagtttgaggcga |  | 30 | aaagaagctctttgccttct |  | 46 | atgccttttcaaatgttcgc |
| 15 | gcaccattaggtaaggacag |  | 31 | tgcttattaaccaggtcgta |  | 47 | ggtcgttcgttagagtgaac |
| 16 | acccgtagaattggagtttg |  | 32 | aattcggcagcatatcgttc |  | 48 | gtcttgatgtgttgcgacaa |
