# Supplementary material for: Resolving noise–control conflict by gene duplication
Source: PLoS Biol. 2019 Nov 22;17(11):e3000289. doi: 10.1371/journal.pbio.3000289 (PMC6874299; doi:10.1371/journal.pbio.3000289)
Supplement: S3 Table — smFISH, single-molecule Fluorescent In Situ Hybridization. (DOCX) [file pbio.3000289.s025.docx]

S3 Table. MSN4 smFISH probes, CAL Fluor Red 590

| PROBE # | PROBE (5'-> 3') |  | PROBE # | PROBE (5'-> 3') |  | PROBE # | PROBE (5'-> 3') |
| --- | --- | --- | --- | --- | --- | --- | --- |
| 1 | ttaggtccgaagactagcat |  | 17 | catgttggtgtggcgataat |  | 33 | ggttgcatctaagctattgt |
| 2 | ttgtttcttgtttgcgtgac |  | 18 | aggtttgtcttgggagaagt |  | 34 | gggtctacgttattgtcgaa |
| 3 | ggctcgttcattatagacga |  | 19 | atattgaaggttctgcctca |  | 35 | taaactgttgatcctgagcc |
| 4 | cagaaacgttggttgtgctc |  | 20 | gtttggtaacctagcatcta |  | 36 | cagaggcattatccgacaac |
| 5 | gctattgttcgcagaattgt |  | 21 | cgttcaatcctgttgtactg |  | 37 | accagaagtcgccaatttag |
| 6 | aaggtatattccggcgaaga |  | 22 | cggcatgaacttcaatgctt |  | 38 | tttggaaagctggtgtaggt |
| 7 | ctatgcggagaatccattga |  | 23 | aaggaagaggtaaccacctc |  | 39 | tggtgagcttgactagtagg |
| 8 | attggagtattagtggcgtc |  | 24 | gttgagtgggtttactgttg |  | 40 | gttgttgatgatgttgagct |
| 9 | cgtattagttgtcgctgtta |  | 25 | gcagtaccttcagggataat |  | 41 | gacgactttcttcttcttgt |
| 10 | ggcggtatcctttaaattca |  | 26 | ggtgaaatggttgatccacg |  | 42 | aaccttaccattgttgttgt |
| 11 | gaatttttccacttccagtt |  | 27 | gctcagagcttgagaaacgt |  | 43 | atagatttccttttccgagg |
| 12 | cattcacgaactgtgcgtta |  | 28 | tttgttataccagagtcctt |  | 44 | tttatcgtagttgttggggt |
| 13 | agcattactgctttgtttct |  | 29 | gctatgggagatgcgattta |  | 45 | cttctcacagtctttacact |
| 14 | cttatcaccaccatttttct |  | 30 | ttaagggcaaagaggcacgc |  | 46 | cggatcttatatgccttttc |
| 15 | gggtggctactgaactatat |  | 31 | gcggaatcagacagaggatt |  | 47 | tacaagcaaaagggcgttcc |
| 16 | ctagttgctccaagtttttc |  | 32 | caccaaaagcatcgtcttca |  | 48 | cgtgctttttgtgagttttt |
