# Supplementary material for: Resolving noise–control conflict by gene duplication
Source: PLoS Biol. 2019 Nov 22;17(11):e3000289. doi: 10.1371/journal.pbio.3000289 (PMC6874299; doi:10.1371/journal.pbio.3000289)
Supplement: S1 Note — (DOCX) [file pbio.3000289.s026.docx]

S1 Note

We note here that there are some differences between our results and the results reported by AkhavanAghdam et al. [1]. In our high temporal resolution data of mRNA sequencing (every 3 minutes for the first hour after stress induction and then every 10 minutes in the next half/one hour), in all the conditions that we tested, there was no fundamental difference in Msn2,4 contribution to the response between the genes DSC2, DDR2 and genes SIP18,TKL2 as was reported (S13 Fig). In all the conditions we tested, these genes show the same kinetics dependency on Msn2 and Msn4. Of note, we measured directly the mRNA and with high temporal resolution, while in the paper the measurements were done by reporter proteins for these 4 genes. We have looked extensively for genes with other dynamic dependency than what we have described, using our extensive time-course data, but could not detect such events. Furthermore, in our time-laps microscopy experiments, we could not detect any differences in the nuclear localization dynamics between Msn2 and Msn4 as was reported by AkhavanAghdam et al. We (as others reported[2–4]) could not detect Msn4-GFP in cells growing in rich media in exponential phase, thus performed our microscopy experiments when introducing stress in higher ODs, when Msn4 was detectable.

It is important to note that we used BY4741 strain for our experiments while AkhavanAghdam et al. used W303 strain, and that the stress perturbations we used in our experiments overlap but identical to the conditions they checked (for example: we haven’t done experiments in Ethanol 3%).

References

1. AkhavanAghdam Z, Sinha J, Tabbaa OP, Hao N. Dynamic control of gene regulatory logic by seemingly redundant transcription factors. Elife. 2016;5. doi:10.7554/eLife.18458

2. Newman JRS, Ghaemmaghami S, Ihmels J, Breslow DK, Noble M, DeRisi JL, et al. Single-cell proteomic analysis of S. cerevisiae reveals the architecture of biological noise. Nature. 2006;441: 840–846. doi:10.1038/nature04785

3. Breker M, Gymrek M, Moldavski O, Schuldiner M. LoQAtE—Localization and Quantitation ATlas of the yeast proteomE. A new tool for multiparametric dissection of single-protein behavior in response to biological perturbations in yeast. Nucleic Acids Res. 2014;42: D726–D730. doi:10.1093/nar/gkt933

4. Huh W-K, Falvo J V., Gerke LC, Carroll AS, Howson RW, Weissman JS, et al. Global analysis of protein localization in budding yeast. Nature. 2003;425: 686–691. doi:10.1038/nature02026
